# Phenotypic and functional translation of *IL1RL1* locus polymorphisms in lung tissue and asthmatic airway epithelium

**DOI:** 10.1172/jci.insight.132446

**Published:** 2020-04-23

**Authors:** Michael A. Portelli, F. Nicole Dijk, Maria E. Ketelaar, Nick Shrine, Jenny Hankinson, Sangita Bhaker, Néomi S. Grotenboer, Ma’en Obeidat, Amanda P. Henry, Charlotte K. Billington, Dominick Shaw, Simon R. Johnson, Zara E.K. Pogson, Andrew Fogarty, Tricia M. McKeever, David C. Nickle, Yohan Bossé, Maarten van den Berge, Alen Faiz, Sharon Brouwer, Judith M. Vonk, Paul de Vos, Corry-Anke Brandsma, Cornelis J. Vermeulen, Amisha Singapuri, Liam G. Heaney, Adel H. Mansur, Rekha Chaudhuri, Neil C. Thomson, John W. Holloway, Gabrielle A. Lockett, Peter H. Howarth, Robert Niven, Angela Simpson, John D. Blakey, Martin D. Tobin, Dirkje S. Postma, Ian P. Hall, Louise V. Wain, Martijn C. Nawijn, Christopher E. Brightling, Gerard H. Koppelman, Ian Sayers

**Affiliations:** 1 Division of Respiratory Medicine, NIHR, Nottingham Biomedical Research Centre, Biodiscovery Institute, University of Nottingham, Nottingham, United Kingdom.; 2 Department of Pediatric Pulmonology and Pediatric Allergology, and; 3 Department of Pathology and Medical Biology, Beatrix Children’s Hospital, University Medical Center Groningen, Groningen Research Institute for Asthma and COPD, University of Groningen, Groningen, Netherlands.; 4 Department of Health Sciences, University of Leicester, Leicester, United Kingdom.; 5 Manchester Academic Health Science Centre, University of Manchester, Manchester, United Kingdom.; 6 The University of British Columbia Center for Heart Lung Innovation, St. Paul’s Hospital Vancouver, Vancouver, British Columbia, Canada.; 7 Division of Epidemiology and Public Health, University of Nottingham, Nottingham, United Kingdom.; 8 Departments of Genetics and Pharmacogenomics, Merck Research Laboratories, Boston, Massachusetts, USA.; 9 Institut universitaire de cardiologie et de pneumologie de Québec, Department of Molecular Medicine, Laval University, Québec, Canada.; 10 University of Groningen, University Medical Center Groningen, Groningen Research Institute for Asthma and COPD, Department of Pulmonary Diseases, and; 11 Department of Epidemiology, Beatrix Children’s Hospital, University Medical Center Groningen, Groningen Research Institute for Asthma and COPD, University of Groningen, Groningen, Netherlands.; 12 Respiratory sciences, University of Leicester, Glenfield Hospital, Leicester, United Kingdom.; 13 Centre for Experimental Medicine, Queens University of Belfast, Belfast, United Kingdom.; 14 Department of Respiratory Medicine, Birmingham Heartlands Hospital and University of Birmingham, Birmingham, United Kingdom.; 15 Institute of Infection, Immunity and Inflammation, University of Glasgow, Glasgow, United Kingdom.; 16 Department of Human Development and; 17 Department of Health & Clinical and Experimental Sciences, Faculty of Medicine and NIH Research (NIHR), Southampton Biomedical Research Centre, University of Southampton, Southampton, United Kingdom.; 18 Respiratory Medicine, Sir Charles Gairdner Hospital, Perth, Australia.; 19 NIHR, Leicester Respiratory Biomedical Research Centre, University of Leicester, Leicester, United Kingdom.

**Keywords:** Cell Biology, Genetics, Asthma, Genetic variation, Molecular genetics

## Abstract

The *IL1RL1* (*ST2*) gene locus is robustly associated with asthma; however, the contribution of single nucleotide polymorphisms (SNPs) in this locus to specific asthma subtypes and the functional mechanisms underlying these associations remain to be defined. We tested for association between *IL1RL1* region SNPs and characteristics of asthma as defined by clinical and immunological measures and addressed functional effects of these genetic variants in lung tissue and airway epithelium. Utilizing 4 independent cohorts (Lifelines, Dutch Asthma GWAS [DAG], Genetics of Asthma Severity and Phenotypes [GASP], and Manchester Asthma and Allergy Study [MAAS]) and resequencing data, we identified 3 key signals associated with asthma features. Investigations in lung tissue and primary bronchial epithelial cells identified context-dependent relationships between the signals and *IL1RL1* mRNA and soluble protein expression. This was also observed for asthma-associated *IL1RL1* nonsynonymous coding TIR domain SNPs. Bronchial epithelial cell cultures from asthma patients, exposed to exacerbation-relevant stimulations, revealed modulatory effects for all 4 signals on *IL1RL1* mRNA and/or protein expression, suggesting SNP-environment interactions. The *IL1RL1* TIR signaling domain haplotype affected IL-33–driven NF-κB signaling, while not interfering with TLR signaling. In summary, we identify that *IL1RL1* genetic signals potentially contribute to severe and eosinophilic phenotypes in asthma, as well as provide initial mechanistic insight, including genetic regulation of *IL1RL1* isoform expression and receptor signaling.

## Introduction

Asthma is a chronic airway disorder characterized by inflammation and widespread variable airflow obstruction that is often reversible, either spontaneously or with treatment (1). Over the years, a significant genetic component to asthma has been identified, and today, over 130 single nucleotide polymorphisms (SNPs) have been reported to be associated with asthma and allergic disease in genome-wide association studies (GWAS) (2–4). One of the most replicated asthma-associated genetic signals is the chromosome 2q12 locus, containing the IL-1 receptor like 1 (*IL1RL1*), *IL18R1*, and IL-18 receptor accessory protein (*IL18RAP*) genes (3, 5–7). *IL1RL1* is predominantly expressed as 2 major splice variants, one of which contains the transmembrane domain encoding the membrane bound receptor (ST2L, IL1RL1-b) that facilitates signal transduction through a Toll–IL-1 receptor (TIR) domain by interacting with/binding to IL-1 receptor accessory protein (*IL1RAP*). This IL-33 receptor is expressed on a number of different cell types relevant to asthma, including inflammatory cells such as T-lymphocytes, innate lymphoid cells, basophils, eosinophils, and mast cells, as well as structural cells such as fibroblasts, endothelial cells, and epithelial cells (8, 9). The other main splice variant encodes the soluble form of the receptor (sST2, IL1RL1-a), which has been detected in both bronchoalveolar lavage fluid and serum in asthma patients. This splice variant is hypothesized to act as a decoy receptor for its ligand, dampening IL-33 activity (10, 11).

The presence of multiple polymorphisms in the *IL1RL1* locus that independently contribute to asthma risk complicates the interpretation of the association signal with the disease (4, 12). Because asthma is known to be a multifactorial and heterogeneous disease (1), we hypothesize that different SNPs within the *IL1RL1* locus drive different subtypes or components of asthma via independent and overlapping functional effects. Disease-associated SNPs may exert their functional effects by changing the protein sequence and/or by affecting levels of gene transcription (expression quantitative trait locus; eQTL). Whereas some SNPs affect gene expression under constitutive conditions (constitutive eQTL), it has recently been shown that the effect of a SNP on gene transcription is sometimes observed only in a specific context, such as diseased conditions (inducible eQTL) (13). We hypothesize that the genetic heterogeneity of the *IL1RL1* locus may be partly due to inducible eQTLs that affect gene transcription in asthma patients but not in healthy controls.

**Figure 1:**
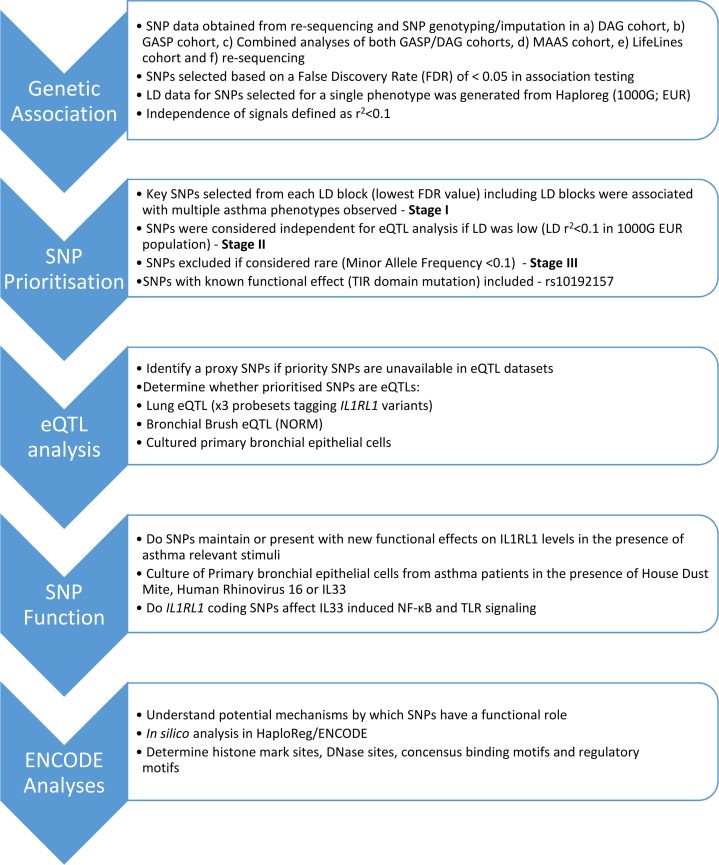
Flow diagram of different stages of investigation carried out in this study. DAG, Dutch Asthma GWAS; ENCODE, Encyclopaedia of DNA Elements; GASP, Genetics of Asthma Severity and Phenotypes; LD, linkage disequilibrium; MAAS, Manchester Asthma and Allergy Study; NORM, Study to Obtain Normal Values of Inflammatory Variables From Healthy Subjects; SNP, single nucleotide polymorphism.

In this study, we set out to extend the association of the *IL1RL1* region polymorphisms with asthma diagnosis and to define the relative contribution of SNPs spanning the association signal to characteristics of asthma defined by clinical and immunological measures. To investigate these hypotheses, we used a step-wise study approach ultimately prioritizing selected association signals for functional characterization (Figure 1). Following detection of known common variation in the locus, we identified coding and noncoding variation through resequencing of the *IL1RL1* locus in 2 European populations of asthma patients in order to provide improved understanding of the genetic variation in the *IL1RL1* region. We subsequently related these SNPs to different asthma subtypes in order to identify key priority SNPs for functional investigation. We tested the presence of specific eQTLs in the lung and bronchial epithelium, with a focus on *IL1RL1* regulation, and we assessed their role in regulating epithelial *IL1RL1* expression after stimulation with known asthma factors implicated in disease exacerbation, such as human rhinovirus 16 (RV-16), a known modulator of IL-33 expression (14); European house dust mite (HDM) extract; and in an artificially IL-33 rich environment. Finally, we performed reductionist functional studies to address the effect of coding SNPs in *IL1RL1* on IL-33–induced signal transduction. In the same system, we investigated the effect of *IL1RL1* coding SNPs on TLR-2 and -4 signaling, both of which have previously been linked to IL1RL1-TLR crosstalk in the context of tolerance (15, 16).

## Results

### Demographics.

For details of all cohorts used in this study, see Supplemental Methods (supplemental material available online with this article; https://doi.org/10.1172/jci.insight.132446DS1).

### Resequencing of the IL1RL1 region.

Resequencing of the chromosome 2 region containing *IL1RL1* in 200 pooled severe asthma patient DNA samples (Genetics of Asthma Severity and Phenotypes [GASP]) and 200 pooled nonasthmatic, nonallergic subject DNA samples (Nottingham Gedling Cohort) identified a total of 4107 variants, of which 1899 were designated as valid variant calls (Supplemental Table 1). Case/control analysis for severe asthma using sequencing allele counts identified 8 variants of interest in severe asthma through meeting criteria of FDR < 0.05, with 3 variants surviving quality control (Supplemental Table 2).

Exon resequencing of the *IL1RL1* gene in an additional 95 asthma patients (Dutch Asthma GWAS [DAG]), carried out to increase our pool of sequenced asthma patients, identified a total of 56 variants covering the gene’s distal and proximal promoter, introns, and exons (Supplemental Table 3).

### Identification of genetic variants associated with subphenotypes of asthma.

Considering the genomic region 400 kb up- and downstream of *IL1RL1* (GRCh37 chr2: 102,527,961–103,368,497) that encompasses all known genetic signals associated with asthma (Supplemental Figure 1), we identified association between 3 SNPs and severe asthma (resequencing analyses; Supplemental Table 2), 130 SNPs and asthma (Lifelines cohort; Supplemental Table 4), 316 SNPs and blood eosinophil levels in a general population (Lifelines; Supplemental Table 5), 4 SNPs and atopy (DAG/GASP; Supplemental Table 6), and 3 SNPs and lung function (forced expiratory volume in the first second [FEV_1_], DAG/GASP; Supplemental Table 7). We did not observe significant associations with lung function (FEV_1_ and FEV_1_/forced vital capacity [FVC]) in Lifelines and Manchester Asthma and Allergy Study (MAAS), nor with blood eosinophils, childhood onset asthma, total IgE levels, or lung function ratio (FEV_1_/FVC) in GASP/DAG.

**Figure 2:**
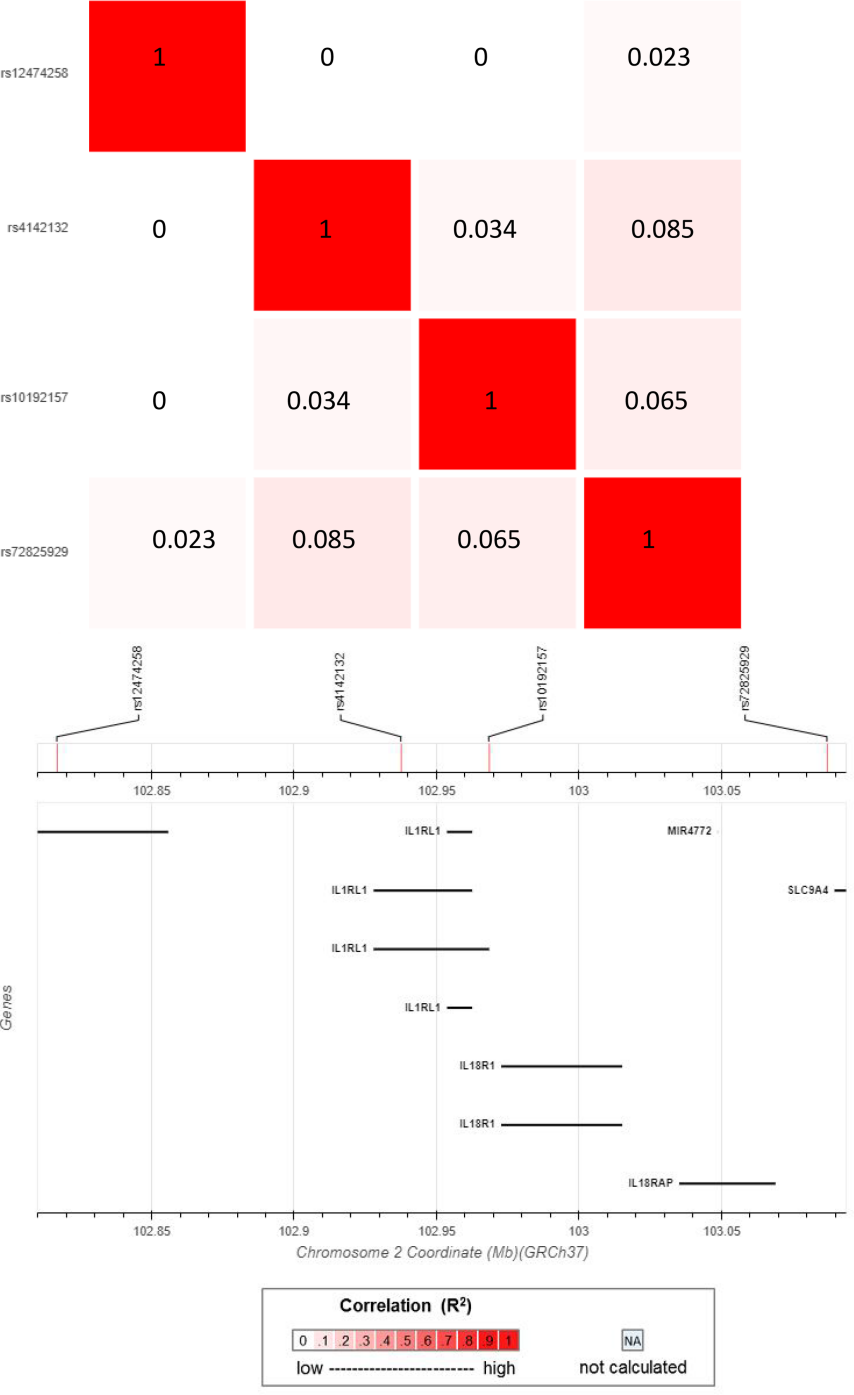
Linkage disequilibrium map of the 4 *IL1RL1* variants identified in Stages 1–3 and selected as SNPs for functional study. Figure identifies the level of linkage disequilibrium between signals identified based on r^2^ values. Image generated using the EUR population of the Phase I cohort of the 1000 genomes study via the LDmatrix tab of the online software tool LDlink 3.6, available at https://ldlink.nci.nih.gov/

We selected 4 signals of association for further functional study by considering (a) significant associations (FDR < 0.05) with asthma subtypes in our genetic association analysis, (b) a minor allele frequency > 0.1 to facilitate subsequent in vitro analysis, (c) independence based on r^2^ < 0.1 in the 1000 genomes EUR population (17), and (d) SNPs that were known to have a functional effect on *IL1RL1* receptor signaling (Figure 1). A detailed description of the SNP selection procedure can be found in the Supplemental Methods. The 3 tagging SNPs for the associated signals presented in this manuscript (Signal A: rs12474258, minor allele frequency [MAF]: 0.40, Asthma (T) OR: 1.20, FDR: 0.049, blood eosinophils (T) β: 0.03, FDR: 0.017; Signal B: rs4142132, MAF = 0.49, FEV_1_ (A) β: –0.07, FDR: 0.029; and Signal C: rs72825929, MAF = 0.10, Severe Asthma (A) χ^2^ statistic: 16.4, FDR: 0.035) span the *IL1RL1* region (Figure 2) and demonstrate association with severe, eosinophilic asthma subtypes (Table 1). For convenience, we will refer in this paper to the signals tagged by these 3 SNPs as Signal A, Signal B, and Signal C. We also included the previously reported *IL1RL1* TIR homology domain coding region variant tagged by rs10192157 (12), referred to as Signal D, for downstream analysis due to its structural changes (Ala433Thr/Gln501Arg/Thr549Ile/Leu551Ser) known to affect/modulate IL1RL1 receptor signaling (18).

### Linkage disequilibrium between selected signals and known asthma signals.

We identified that, of our 3 selected signals (discounting Signal D, [rs10192157], which was selected as a functional variant based on literature), in the 1000 Genomes EUR population, only Signal B (rs4142132) is in linkage disequilibrium (LD) with a previously reported asthma association signal (LD r^2^ > 0.5; Supplemental Figure 2). Signal B includes reported asthma SNPs (rs11685480 [r^2^ = 1 with rs4142132], rs12479210 [r^2^ = 0.54], and rs1420101 [r^2^ = 0.52]). Our remaining selected signals (rs12474258 and rs72825929) show low LD (r^2^ = 0.1), with SNPs previously associated with asthma diagnosis. For a full visualization of the LD patterns to our 3 selected SNPs, refer to Supplemental Figure 2.

### Association testing of previously reported asthma signals with asthma subphenotypes in our cohorts.

A literature search identified 42 studies highlighting 19 reported SNPs associated with asthma (Supplemental Table 8). Based on r^2^ < 0.1, these associations represent a single, independent signal. We were able to replicate association with asthma diagnosis in the Lifelines cohort for 2 known asthma-associated SNPs rs13431828 (OR: 1.36, FDR: 0.040) (19–21) and rs10173081 (OR: 1.36, FDR: 0.040) (6, 22), which represent the same genetic signal (r^2^ = 1, 1000 Genomes EUR population). The reported asthma risk alleles for rs13431828 (C) and rs10173081 (C) are consistent with the rs12474258 risk allele (C) identified in the current study (i.e., our signal shows the same direction of effect). We did not observe any additional association with these 19 reported SNPs to any of the phenotypes tested in any cohort (FDR < 0.05). Investigation to see if the 19 asthma SNPs also associate to blood eosinophil levels in both our cohorts and the literature; we identified a degree of overlap, confirming the association of Signal A (with which the overlapping SNPs are associated) with asthma and blood eosinophil levels (Supplemental Table 8).

### Selected signals act as eQTLs for membrane and soluble IL1RL1 encoding transcripts in lung tissue.

To assess functional consequences of the selected signals, we first performed an eQTL analysis in lung tissue utilizing array data with *IL1RL1* isoform–specific probes. We find that 3 of the 4 signals (Signals B, C, and D tagged by rs142008 [proxy for rs4142132], rs11690532 [proxy for rs72825929], and rs10192157, respectively; Table 2) act as eQTLs for *IL1RL1* in whole lung tissue (Table 3). The FEV_1_ risk allele for Signal B (proxy rs1420088 [C] allele) was shown to be associated with attenuated levels of *IL1RL1* mRNA of isoforms encoding both the soluble (IL1RL1-a) and transmembrane (IL1RL1-b) protein. The asthma risk allele for Signal C (proxy rs11690532 [C]), however, was associated with elevated *IL1RL1* mRNA expression (of both transcripts in a combined and independent assay). The asthma risk allele for Signal D (rs10192157 [C]) was associated with lower expression of the transcript encoding the soluble isoform but did not show association with the transcript encoding the transmembrane protein. In summary, these data show that 3 of our prioritized signals are eQTLs for *IL1RL1* in lung tissue; however, the 2 asthma-associated risk alleles have opposite directions of effect on *IL1RL1* mRNA expression in lung tissue. Interestingly, Signal A — associated with asthma and blood eosinophils — did not demonstrate any *IL1RL1* eQTL association in lung tissue. The strongest effect estimate was observed with the *IL1RL1* isoform encoding the soluble protein, showing a 10-fold increase over the transmembrane isoform in the presence of the respective risk alleles of Signals B and C (rs1420088 and rs11690532; Table 3).

### Association between IL1RL1 SNPs and baseline IL1RL1 expression in cultured bronchial epithelial cells from asthma patients.

**Figure 3:**
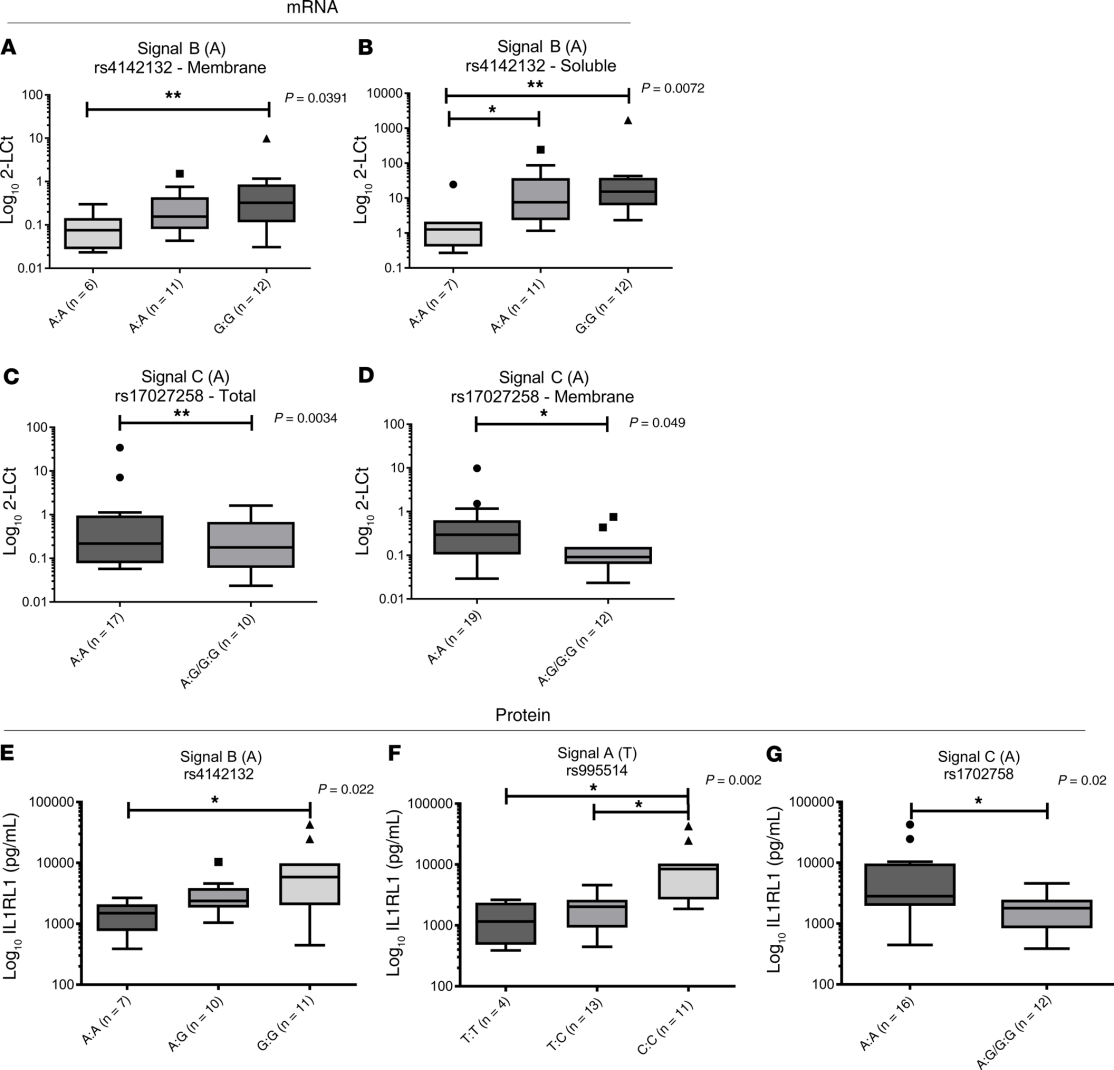
Baseline *IL1RL1* mRNA and soluble IL1RL1 protein levels are driven by SNPs in cultured human bronchial epithelial cells. (**A** and **B**) In cultured HBECs, the lower level of *IL1RL1* soluble and transmembrane mRNA can be observed in carriers of the risk allele (A) for lower lung function (FEV_1_) for Signal B (rs4142132; **A** [AA, *n* = 6; AG, *n* = 11; GG, *n* = 12] and **B** [AA, *n* = 7; AG, *n* = 11; GG, *n =* 12]; *P* < 0.05). (**C** and **D**) Increased levels of total and transmembrane *IL1RL1* mRNA was observed for carriers of the asthma risk allele (A) in Signal C (rs17027258, proxy for rs72825929; **C** [AA, *n* = 17; AG/GG, *n* = 10] and **D** [AA, *n* = 19; AG/GG, *n* = 12]; *P* < 0.05). (**E–G**) Changes in mRNA levels were reflected in soluble IL1RL1 protein levels in matched cellular supernatants (**F** [TT, *n* = 7; TC, *n* = 10; CC, *n* = 11] and **G** [AA, *n* = 16; AG/GG, *n* = 12]; *P* < 0.05); in Signal A, carriers of the asthma risk/elevated blood eosinophil levels allele of SNP rs995514 (proxy for rs12474258) (T) presented with lower levels of IL1RL1 soluble protein (**E** [TT, *n* = 4; TC, *n* = 13; CC, *n* = 11]; *P* = 0.002). However, this was not observed at the RNA level (Supplemental Figure 3). Statistics were run using Mann-Whitney *U* test (**C**, **D**, and **G**) or Kruskal-Wallis test (**A**, **B**, **E**, and **F**), as relevant. Data are represented by Tukey box and whisker plots, where the box covers data from the 25th to the 75th percentiles, with the center line denoting the median of the data. Whisker plots identify the interquartile range as determined by the Tukey method, with resulting outlier data displayed as distinct points outside the whiskers.

To determine the effect of the 4 signals on *IL1RL1* expression in cultured bronchial epithelial cells (BECs), we examined the effect of SNPs on baseline expression of *IL1RL1* mRNA isoforms and soluble IL1RL1 (IL1RL1-a) protein in human BECs (HBECs) isolated from asthma patient donors and cultured in vitro (Figure 3 and Supplemental Figures 3 and 4). We observed that Signal B, tagged by the *IL1RL1* intronic variant rs4142132, had an effect on the mRNA levels of the transcripts encoding soluble and membrane IL1RL1 isoforms in HBECs cultured in vitro. The presence of the risk (A) allele associated with a lower FEV_1_ resulted in lower levels of both *IL1RL1* mRNA isoforms (*P* < 0.05) (Figure 3, A and B), an effect mirrored in the whole lung tissue. These results were confirmed at the protein level, where levels of soluble IL1RL1 in HBEC supernatants were lower in carriers of the (A) allele (*P* < 0.01) (Figure 3E).

The allele associated with severe asthma at Signal C (rs17027258 [A]) was associated with elevated *IL1RL1* mRNA levels of the transcripts encoding transmembrane IL1RL1 but not with those encoding the soluble isoform in HBECs (*P* < 0.05) (Figure 3, C and D), in agreement with the direction of effect observed in lung tissue. At the protein level, the risk allele (A) was associated with elevated soluble IL1RL1 levels in cellular supernatants (Figure 3G).

In HBECs, as opposed to no effect in whole lung tissue, the asthma risk (T) allele for Signal A (tagged by rs995514) also associated with elevated blood eosinophil levels, resulted in a lower levels of soluble IL1RL1 protein, but had no effect on mRNA isoforms (Figure 3F and Supplemental Figures 3 and 4). For Signal D (rs10192157), no effect was observed on either *IL1RL1* mRNA or protein levels (Supplemental Figures 3 and 4).

### Effect of asthma-relevant stimuli on IL1RL1 expression.

**Figure 4:**
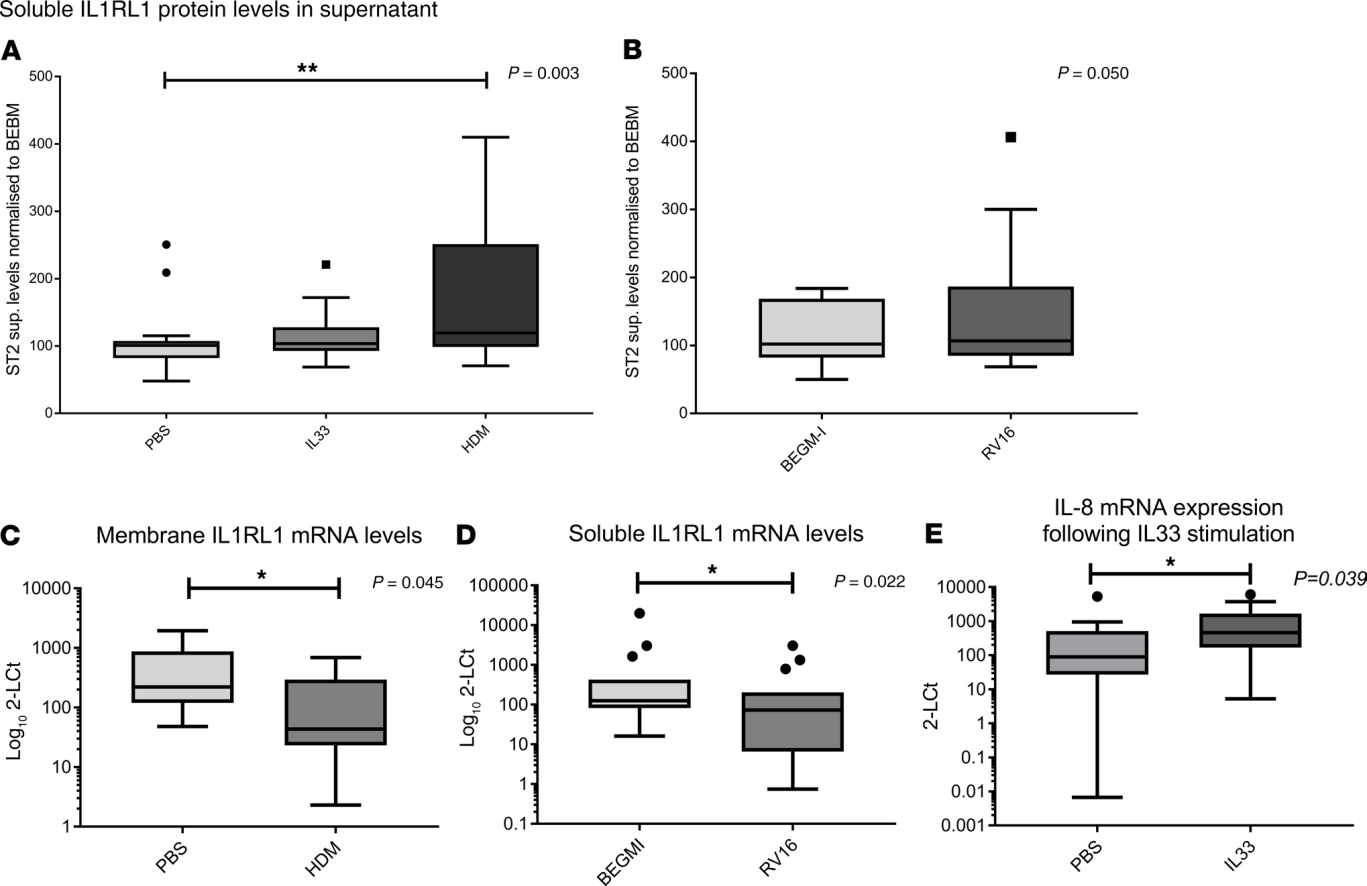
Asthma relevant microenvironments modulate *IL1RL1* mRNA levels and soluble IL1RL1 protein levels in bronchial epithelial cells isolated from asthma patients and cultured in vitro. (**A**) Stimulation of cells with 50 μg/mL house dust mite (HDM) for 24 hours resulted in increased release of soluble IL1RL1 into the cellular supernatant (*P* = 0.003, *n* = 18). (**B**) RV-16 (MOI: 1) stimulation for 24 hours did not significantly influence IL1RL1 protein release in the cell supernatants (*P* = 0.05, *n* = 18). (**C** and **D**) HDM stimulation resulted in a 3.5-fold reduction of membrane IL1RL1 mRNA (**C**) (*P* = 0.045, *n* = 15), while stimulation with RV-16 (MOI: 1) for 24 hours reduced soluble IL1RL1 mRNA levels 4.4-fold (**D**) (*P* = 0.022, *n* = 15). (**E**) IL-33 stimulation did not alter IL1RL1 protein or mRNA levels; however, it did induce IL-8 mRNA, demonstrating cell activation (*P* = 0.039, *n* = 18). Statistics were run using Mann-Whitney *U* test (**B–E**) or Kruskal-Wallis test (**A**), as relevant to the data. Data are represented by Tukey box and whisker plots, where the box covers data from the 25th to the 75th percentiles, with the center line denoting the median of the data. Whisker plots identify the interquartile range as determined by the Tukey method, with resulting outlier data displayed as distinct points outside the whiskers.

Next, we considered the possibility that disease state and/or relevant microenvironmental triggers may regulate IL1RL1 expression in a SNP-dependent fashion. Therefore, we investigated the effect of asthma-relevant stimulations of cultured HBECs obtained from asthma subjects on *IL1RL1* expression in carriers and noncarriers of phenotype-associated alleles (i.e., inducible eQTLs). Prior to stratification, we observed an increase in soluble IL1RL1 protein levels in cell supernatants following stimulation with HDM for 24 hours (*P* < 0.01); however, no change was observed with either RV-16 or with IL-33 stimulation (*P* > 0.05) (Figure 4, A and B). Conversely, stimulation with HDM reduced membrane *IL1RL1* mRNA levels 3.5-fold (Figure 4C, P** < 0.05), while RV-16 stimulation reduced soluble *IL1RL1* mRNA expression 4.5-fold (Figure 4D, P** < 0.05). No alterations in *IL1RL1* mRNA were observed in response to IL-33; however, a response to IL-33 was confirmed using IL-8 mRNA levels as an outcome (Figure 4E).

### IL1RL1 variation has an impact on IL1RL1 regulation in response to asthma-relevant stimuli.

**Figure 5:**
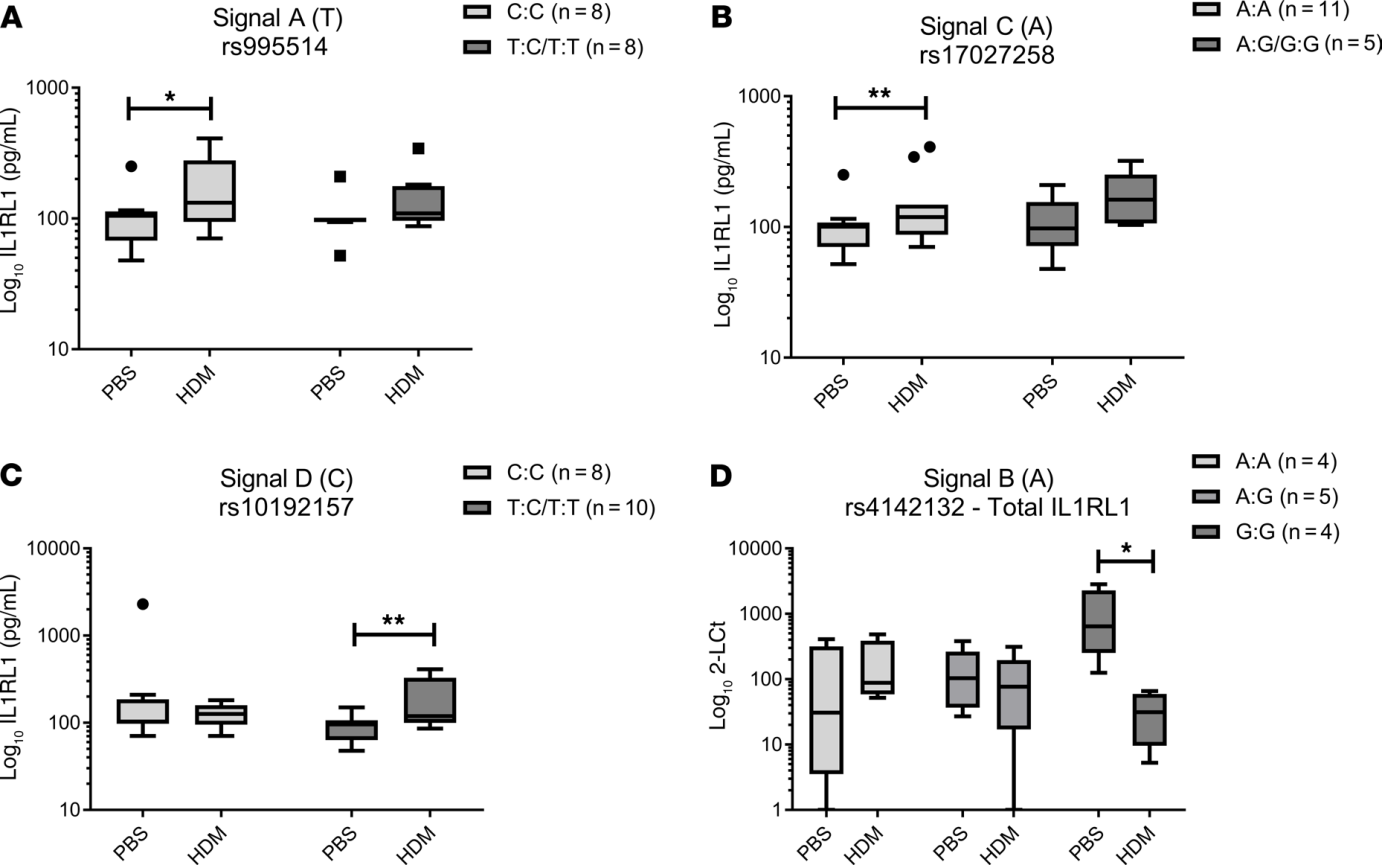
SNPs regulate *IL1RL1* mRNA and protein expression levels in response to asthma-relevant microenvironments. (**A–C**) Increased release of IL1RL1 protein in response to HDM was present in 3 of our 4 selected signals: Signal A (rs995514; proxy for rs12474258) for the protective allele for asthma and elevated blood eosinophils (C) (*P* < 0.05), Signal C (rs17027258; proxy for rs72825929) risk allele for severe asthma (A) (*P* < 0.01), and Signal D (rs10192157) for the protective allele for asthma (T) (*P* < 0.05) (**A** [CC, *n* = 8; TC/TT, *n* = 8], **B** [AA, *n* = 11; AG/GG, *n* = 5], and **C** [CC, *n* = 8; TC/TT, *n* = 10], respectively). (**D**) Decreased levels of total *IL1RL1* mRNA in response to HDM is present only in Signal B (rs4142132) for carriers of the allele protective for reductions in lung function (FEV_1_) (G) (AA, *n* = 4; AG, *n* = 5; GG, *n* = 4; *P* < 0.05). Statistics were run using a Kruskal-Wallis test. Data are represented by Tukey box and whisker plots, where the box covers data from the 25th to the 75th percentiles, with the center line denoting the median of the data. Whisker plots identify the interquartile range as determined by the Tukey method, with resulting outlier data displayed as distinct points outside the whiskers.

Stratification based on our 4 selected signals identified that HDM-driven effects on *IL1RL1* expression were genotype dependent, with effects observed for all 4 selected SNPs (Figure 5 A–D, Supplemental Figures 5 and 6).

In Signal A (tagged by rs995514), there was a modest increase in the level of soluble IL1RL1 protein (1.63-fold) in the presence of the asthma protective allele (C) (Figure 5A). In Signal C (tagged by rs17027258), presence of the severe asthma risk allele (A) identified modest elevation in IL1RL1 soluble protein expression after HDM stimulation (1.5-fold; *P* < 0.01) (Figure 5B). The largest response to HDM was observed in the functionally relevant TIR domain haplotype (Signal D [tagged by rs10192157]). Here, the presence of the protective allele (T) was associated with a 2-fold increase in soluble IL1RL1 protein after HDM stimulation (Figure 5C). No effect was observed on IL-33 stimulation (Supplemental Figure 5).

At the mRNA level, no apparent effects were observed for Signals A, C, and D, in contrast to the observed effects at the protein level (Supplemental Figure 6). However, in Signal B, total *IL1RL1* mRNA expression was reduced (31.73-fold) in response to HDM but only in cells carrying the allele (G) associated with higher lung function (FEV_1_) (Signal B, Figure 5D, P** < 0.05). However, it is important to note that the baseline levels of total *IL1RL1* mRNA in GG carriers were significantly higher than that for both AA and AG genotype carriers, in keeping with the findings reported above (Figure 3, A and B).

### IL1RL1 coding region variants associated with asthma influence signaling.

**Figure 6:**
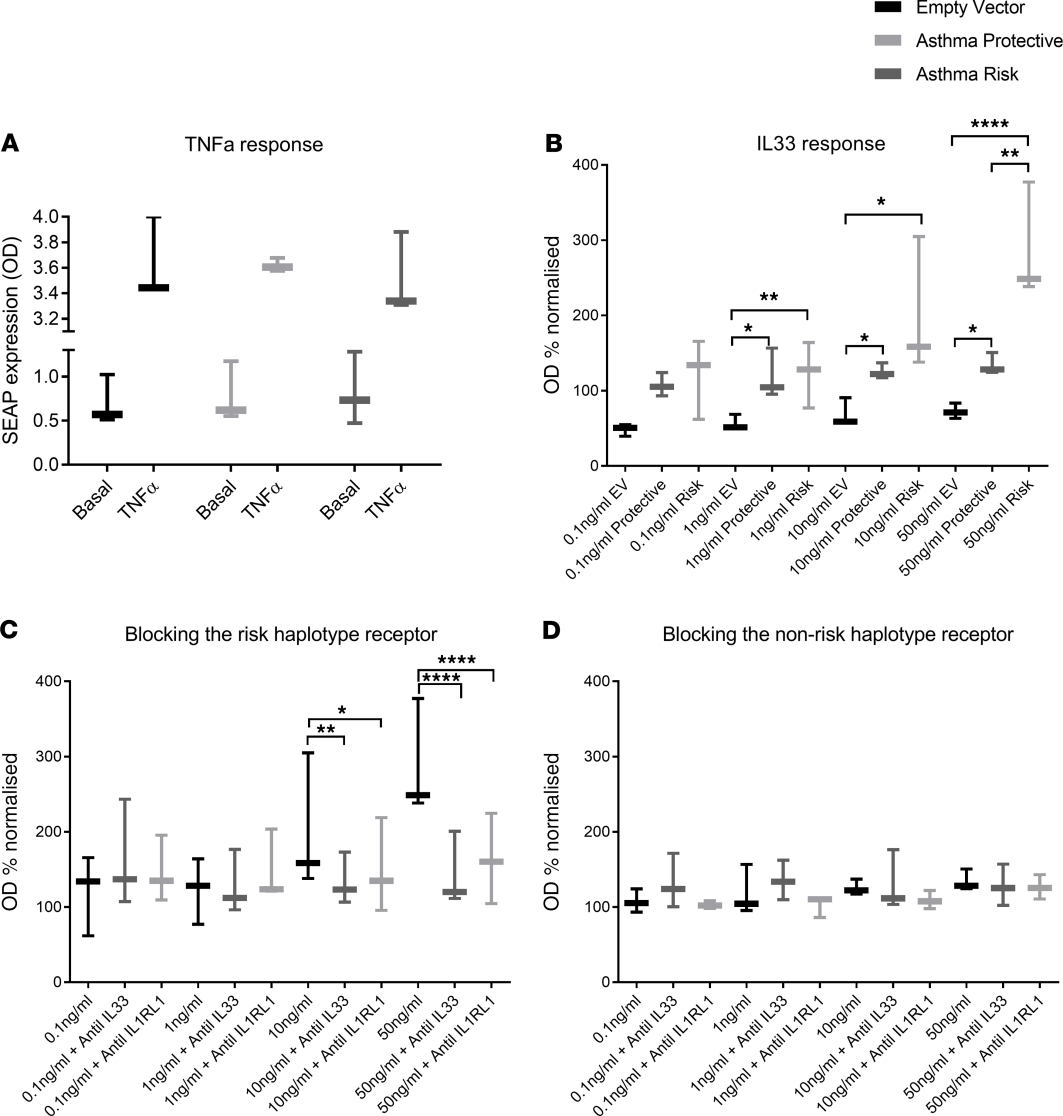
Functional analyses of the *IL1RL1* TIR risk haplotype in an in vitro reductionist model identifies an exaggerated response to IL-33 that is more amenable to anti–IL-33/IL1RL1 treatment. Transient transfection of HEK–NF-κB–SEAP reporter cells with *IL1RL1* containing the 2 TIR domain polymorphism haplotypes provides a platform to identify differential NF-κB signaling. (**A**) Cells transfected with empty vector, *IL1RL1* containing the asthma risk haplotype (Ala433/Gln501/Thr549/Leu551), or IL1RL1 containing the protective haplotype (Thr433/Arg501/Ile549/Ser551) have the same capacity to signal via the NF-κB pathways in response to 10 ng/mL TNF-α. (**B**) The presence of the IL1RL1 receptor carrying the asthma risk haplotype identified a 2-fold and 3-fold increase in signaling on stimulation with 10 ng/mL and 50 ng/mL of human recombinant IL-33, respectively, whereas an attenuated response was observed in the protective haplotype. (**C**) The response induced by 50 ng/mL IL-33 in the risk haplotype was amenable to blocking using either 10 μg/mL anti–IL-33 or anti-IL1RL1 leading to an antiinflammatory effect. (**D**) The effect of blocking IL-33 induced inflammation by anti–IL-33 or anti-IL1RL1 was minimal in carriers of the protective TIR domain haplotype. *P < 0.05, **P < 0.01, ****P < 0.0001. *n* = 3 for all experiments. Statistics were run using a Kruskal-Wallis test. Data are represented by Tukey box and whisker plots, where the box covers data from the 25th to the 75th percentiles, with the center line denoting the median of the data. Whisker plots identify the interquartile range as determined by the Tukey method, with resulting outlier data displayed as distinct points outside the whiskers.

We next tested functional effects of the membrane *IL1RL1* TIR domain haplotype that are tagged by Signal D. These haplotypes encode *IL1RL1* proteins that present with a 4–amino acid change in the intracellular TIR signaling domain (Ala433Thr/Gln501Arg/Thr549Ile/Leu551Ser) (12). The potential functional effects include the following: (a) *IL1RL1-b* coding region variants determine the magnitude of signaling response downstream of IL-33 (23) and (b) *IL1RL1* haplotypes determine the antiinflammatory effects of anti–IL-33 and anti-IL1RL1 monoclonal antibodies (23). A reductionist recombinant cell line model with a fixed genetic background was used to facilitate these analyses. HEK-Blue-SEAP cells transfected with empty vector or 1 of the 2 *IL1RL1* mRNAs encoding the alternative TIR domain IL1RL1 proteins, which demonstrated the same capacity to signal via NF-κB following TNF-α stimulation (Figure 6A). Escalating doses of recombinant IL-33 were able to induce NF-κB signaling, in a dose dependent manner, in the 2 cells lines containing the IL1RL1 protein (Figure 6B). Cells carrying the asthma risk haplotype (Ala433/Gln501/Thr549/Leu551) tagged by the Signal D SNP rs10192157 (CC) demonstrated a 2.9-fold induction at the highest dose of IL-33 (50 ng/mL) in this cell system, which was significantly higher than the modest activity observed for the asthma protective TIR domain haplotype protein (Thr433/Arg501/Ile549/Ser551) (1.3-fold) (Figure 6B). Additionally, cells carrying the asthma risk haplotype and stimulated with 50 ng/mL IL-33 were more amenable to the antiinflammatory effects of blocking either IL1RL1 or IL-33 using monoclonal antibodies compared with the alternative haplotype, where blocking antibodies had a minimal effect on reducing NF-κB signaling (Figure 6, C and D).

### IL1RL1 coding region variants associated with asthma do not modify TLR signaling.

IL1RL1 signaling is thought to modify TLR-2 and -4 activation (15, 16); therefore, we hypothesized that the *IL1RL1* TIR domain haplotype variation may influence this relationship. For these experiments, we used *ILR1L1* overexpression vectors in transfected cells whose functionality was confirmed by the capacity to induce ERK1/2 activation after IL-33 exposure (Supplemental Figure 7). We did not observe an effect of overexpression of the 2 different *IL1RL1* exon 11 haplotypes on the sensitivity of HEK-Blue cells to TLR2-induced NF-κB activity after stimulation using a dilution series of Pam3Cys (Supplemental Figure 8A). We also did not observe an *IL1RL1* exon 11 haplotype–driven effect on TLR4-induced NF-κB activity in HEK-Blue cells after stimulation with a dilution series of LPS (Supplemental Figure 8B). These studies show that, while the *IL1RL1* exon 11 haplotype regulates sensitivity to IL-33 (Figure 6), the proposed regulatory function of IL1RL1-b on TLR2 or TLR4 signaling (15, 16) could not be confirmed (Supplemental Figure 8).

### Bioinformatic analyses of prioritized signals using ENCODE.

We investigated each signal including SNPs in LD defined by r^2^ > 0.8 for potential functional effects using the Encyclopaedia of DNA Elements (ENCODE) resource via HaploReg (Supplemental Table 9). Two of the investigated LD blocks tagged by Signals A and B (rs12474258 and rs4142132) were found to have multiple SNPs positioned in enhancer histone mark sites, with DNase hypersensitivity sites also identified within the Signal B region (rs4142132), a region where protein binding consensus sequences exist and affect regulatory motifs. Motifs changed in Signals A and B included cell type–specific transcription factors such as GATA-2, active in mast cells and basophils (24) and positively regulating *IL1RL1* expression (25), and FOXA1/2, active in HBECs (26). Similarly, the functional SNP rs10192157 (Signal D) altered Gfi1 transcription factors linked to type 2 inflammation and a regulator of IL1RL1 expression (27). There was also generic activation–induced transcription factors such as Fos/Jun (AP-1), NF-κB, and cEBP/p300 found to be bound to these motifs. This highlights potential functional effects for 3 of our 4 selected signals of association on both cell type–specific and ubiquitous regulation of *IL1RL1* expression (Supplemental Table 9). No SNPs were identified to be in LD for Signal C (rs72825929), and no enhancer histone mark sites, DNase hypersensitivity, or protein motif binding were identified for this SNP. However, rs72825929 modifies the Sox (sex-determining region Y [Sry] box–containing) family of transcriptional factors, which have been shown to be involved in lung organogenesis, with Sox2 in particular playing a crucial role in the proliferation and differentiation of respiratory epithelial, trachea, airway branching, and Clara cells (28).

## Discussion

### Main findings.

We set out to extend our understanding of the *IL1RL1* locus in asthma, one of the most reproducible association signals identified to date, with a particular focus to the contribution of the *IL1RL1* gene. We provide insight into the nature of the genetic association with clinical and immunological features of asthma and the mechanistic underpinnings of these associations with respect to *IL1RL1* expression and activity. In this multiple-cohort study, we extend a priori evidence that genetic variation in the *IL1RL1* region is important for asthma susceptibility and blood eosinophil counts. In particular, we identify that a functional haplotype consisting of several polymorphisms encoding 4 amino acid changes (increased asthma risk, Ala433/Gln501/Thr549/Leu551) and present in ~50% of the European population enhanced NF-κB activity after IL-33 stimulation. This has potentially important implications for targeted asthma therapies, where expression of the risk haplotype could influence therapeutic efficacy. Functional analyses demonstrated that 3 of our 4 independent signals of association, Signals B, C, and D — tagged by SNPS rs4142132, rs72825929, and rs10192157 — are eQTLs for *IL1RL1* in lung tissue. These regulatory roles were retained in cultured primary HBECs at both the mRNA and protein levels. Interestingly, although not an eQTL in the whole lung data set, Signal A (rs12474258) was an eQTL in cultured BECs for soluble IL1RL1 protein. Thus, our data suggest cell type specificity of eQTL for *IL1RL1*, suggesting that specific subtypes of asthma may be driven by different cell types in different patients. Therefore, different asthma subtypes may be compartment specific, where specific cell types such as lung structural, inflammatory, and isolated immature basal epithelial cells may predominate.

Interestingly, there was a lack of eQTL effects observed in bronchial biopsies and bronchial brushes taken from controls without respiratory diseases, while we did observe eQTLs in cultured primary HBECs of asthma patients, compatible with the context dependency of eQTLs. We therefore investigated whether the selected SNPs act in such a context-dependent manner (i.e., inducible eQTLs), through functional studies. We identified that all of our signals of interest were able to modulate response to HDM, a common allergen associated with asthma and allergies, on *IL1RL1* expression in HBECs. These data provide a potential link between *IL1RL1* genetic variants and *IL1RL1* regulation of IL-33 inflammation in HDM-induced type 2 immune responses in the lung, where the IL-33/IL1RL1 axis has been shown to be involved (29, 30). Finally, we also investigated *IL1RL1* coding region variation and established that cells carrying the *IL1RL1* TIR domain asthma risk haplotype (Signal D) presented with an exaggerated inflammatory response to IL-33 that is more amenable to the antiinflammatory effects of either anti–IL-33 or anti-IL1RL1 monoclonal antibodies. This has implications for the targeting of the IL-33/IL1RL1 axis inhibitors to a subset of patients of a specific genotype likely to gain the greatest clinical benefit and is highly relevant, with multiple pharmaceutical companies developing anti–IL-33/IL1RL1 approaches for the treatment of asthma.

### Genetic associations.

The locus on chromosome 2q12, which includes the *IL1RL1* gene, has shown a significant replicated association with asthma (5, 7, 18, 20, 22) or asthma-relevant traits such as childhood asthma (21, 31, 32), childhood asthma with exacerbation (31), severe asthma (33, 34), asthma with hay fever (35), type 2 inflammation in asthma (36), and blood eosinophil counts (20). Sentinel SNPs identified in these association studies span the 2q12 region, covering multiple genes, with evidence based on linkage disequilibrium pointing to independent association signals (20).

In order to characterize further the contribution of genetic variants to features of asthma, we carried out association testing across the Lifelines/GASP/DAG/MAAS cohorts. This identified that chromosome 2q12 region variation is associated with blood eosinophil numbers (Signal A) and lung function (FEV_1_) (Signal B). We also investigated SNPs previously associated with asthma or asthma-related traits in the region, with 2 of these SNPs, rs13431828 and rs10173081, being associated with asthma diagnosis in our Lifelines analysis (Supplemental Table 2). We also confirmed phenotypic overlap between asthma and blood eosinophil counts in Signal A by investigating 19 SNPs, previously associated with asthma, in this region in LD with Signal A. Significant overlap between these SNPs and SNPs associated with eosinophilia counts in our cohorts and in the literature was observed (Supplemental Table 8). To complement the investigation of the genetic architecture of the locus, we completed next-generation sequencing analyses and identified 4107 variants spanning the region, of which 3 met criteria for association with severe asthma in case/control analyses (FDR < 0.05) and survived quality control, including sentinel variant rs72825929 (A/G [5′ to *SLC9A4*]), Signal C. Although limited by small population numbers and pooled analysis, additional support for the association of Signal C with asthma was determined through previously published GWAS, where association was shown with all asthma (rs11690532) (5) and pulmonary function (rs17027258/rs11690532; FEV_1_ and FEV_1_/FVC) (37). More importantly, Signal C was also associated with moderate-to-severe asthma in the largest GWAS of this phenotype published to date, to our knowledge (rs72825929; β: 0.11, *P* = 0.0016, Allele A) (33). In Lifelines, rs72825929 was associated with blood eosinophil counts in the general population ([G] β: 0.05, SE: 0.015, FDR: 0.03, AF general population [G]: 0.10) but not with the other studied phenotypes. Based on these analyses, we selected 3 SNPs that identify key signals of association spanning the region for functional analyses and also included TIR domain SNP rs10192157 (Signal D) from the literature, giving a total of 4 signals for functional follow-up. Overall, these data complement and extend the accumulating data, suggesting a role for the IL-33/IL1RL1 axis that is genetically determined in T2-driven inflammation (36, 38) and eosinophilic asthma (20). Taken together, our data suggest that there are multiple independent genetic signals in the *IL1RL1* locus that may be particularly important in driving severe, eosinophilic asthma phenotype with reduced lung function, with limited evidence for genetic variants driving other features of asthma such as atopy, total IgE levels, and age at onset in these cohorts.

### eQTLs.

Previous work has established that genetic variation in the *IL1RL1* locus can act as eQTLs for *IL1RL1* mRNA, as methylation QTLs in WBCs and as protein QTL (pQTL) for serum levels of soluble IL1RL1 (13, 21, 23, 38–40). In this study, we specifically investigated the functional effects of our 4 priority signals in (a) lung tissue, (b) airway epithelial brush samples, and (c) cultured HBECs at baseline and in the presence of asthma-relevant stimuli. Lung tissue eQTL identified 3 of the 4 selected signals as eQTLs for *IL1RL1*, with differential SNP effects observed for membrane and soluble isoforms. Interestingly, the presence of the asthma risk allele for the 2 asthma selected *IL1RL1* signals (Signals A and C) presented with contradictory effects of *IL1RL1* mRNA transcripts in lung tissue, suggesting that these 2 independent signals may have different roles/functionality with regard to *IL1RL1* and asthma. The effect size of the associations was greatest for the soluble *IL1RL1* mRNA, suggesting that *IL1RL1* eQTL effects are more likely to translate to functional effects through regulation of soluble rather than membrane *IL1RL1* levels, with the exception of the TIR domain–effecting SNP rs10192157 (Signal D). This latter SNP has also been identified as an eQTL for *IL1RL1* in nonstructural cells (41). Our data are in good agreement with the lung eQTL data found in the GTEx database (https://gtexportal.org/home/) (42). However, when comparing our 3 eQTL signals with the strongest *IL1RL1* eQTL signals in this data set, a near-perfect LD pattern (r^2^ ≥ 0.98) was only observed for Signal B, which may suggest that the other 2 eQTL signals (Signals C and D) may be LD shadows of the true causative eQTL variant. On the other hand, our data are further supported by being in good agreement with previous work examining genetic variants that are associated with soluble *IL1RL1* mRNA (36) and serum levels of soluble IL1RL1 (23). More specifically, Signals A–D have all recently been associated with plasma IL1RL1 levels (43). Searching for our 4 signals on Open target genetics (https://genetics.opentargets.org/, accessed June 18, 2019), we identified that the risk allele in Signal A (T) was associated with elevated *IL1RL1* levels in blood plasma (β: -0.202; *P* = 8.3 × 10^–16^), which contrasts to the lack of eQTL reported in our lung and biopsy/brush eQTL data sets. The remaining eQTLs in plasma extracted from whole blood provided additional insight (e.g., the risk allele for Signal B [A] was associated with lower levels of plasma *IL1RL1* [rs10179654, β: –0.85, *P* = 3.00 × 10^–391^]) (43), which supports our findings and the concept that blood levels of soluble ST2 may be driven by epithelial produced protein (36). The risk allele for Signal C (A) was associated with elevated levels (β: –0.469, *P* = 5.5 × 10^–34^), and the risk allele of Signal D (C) was associated with lower levels of *IL1RL1* (β: –0.288, *P* = 1.7 × 10^–30^) in good agreement with our data across data sets.

Importantly, our data extend this work by offering a comprehensive analysis of all *IL1RL1* transcripts (total, membrane, and soluble IL1RL1 encoding mRNA) and extends the recently suggested concept that asthma risk alleles essentially lead to a decrease in soluble IL1RL1, and this lack of decoy receptor diminishes the ability to mitigate the effects of IL-33 (36). Importantly, these analyses highlight multiple signals spanning the IL1RL1 locus that can regulate IL1RL1 and contribute to disease mechanisms via modulation of IL1RL1 receptor levels.

We did not observe SNP eQTL effects in the biopsy and bronchial brush data sets generated from tissue samples obtained from volunteers without respiratory diseases, which is in contrast to a recent study that identified 3 *IL1RL1* SNPs (rs12712135; tagged by Signal B; rs1420088 [r^2^ = 0.98], rs1041973 and rs10185897), which showed association with membrane *IL1RL1* mRNA in bronchial brush samples from asthma patients (38). This can potentially be explained due to differences in sample cell composition and by the fact that the eQTL was run in a nonasthmatic population in our analyses, which would indicate that the eQTL effects are disease specific. To provide greater insight, we therefore completed reductionist eQTL analyses in cultured HBECs isolated from asthma patients. Here, we confirmed the signals tagged by Signals B and C (rs4142132 and rs72825929) as eQTLs, where the observed direction of effect for both signals complemented those seen in our lung tissue data base. Interestingly, we demonstrated that the asthma protective allele (C) for Signal A (rs995514) was an eQTL in cultured epithelial cells, being associated with elevation in soluble protein levels. In contrast, no eQTL could be identified for Signal D (rs10192157). These data provide an additional indication that *IL1RL1* SNPs act as an eQTL in a tissue- and cell type–specific manner, potentially with inflammatory cells contributing to the lung tissue findings (e.g., mast cells, basophils, Th2 cells, ILC2s, and eosinophils). These data do not support the hypothesis that presence of asthma risk alleles leads to reduced soluble IL1RL1 to act as a decoy, limiting the mitigation of the biological effects of IL-33; however, they do suggest a complex effect of genetic signals on membrane and soluble IL1RL1 levels that is cell, tissue, and context dependent. For example, we hypothesize that changes in the IL1RL1 TIR signaling domain structure, — driven by the variation in Signal D, which we have shown drives increased receptor signaling — may cause a negative feedback loop that attenuates IL1RL1 and concurrent soluble IL1RL1 expression.

### Inducible eQTLs.

We tested for inducible eQTLs of our priority signals by culturing HBECs in the presence and absence of RV-16, HDM, and human recombinant IL-33. In general, RV-16–stimulated HBECs showed a decrease in soluble IL1RL1 mRNA; however, no SNP-specific effects were observed, suggesting a limited role of these eQTLs in regulating RV-16–driven effects on soluble *IL1RL1* mRNA.

When considering the aeroallergen HDM, another relevant environmental agent involved in allergic asthma, our data identified that HBECs release soluble IL1RL1 in response to HDM; however, this response was accompanied by a decrease in membrane IL1RL1 mRNA. This is especially relevant when considering that elevations of circulating IL-33 on HDM stimulation have been reported in mouse models (44, 45), with attenuated HDM-induced airway hyperresponsiveness in IL1RL1-KO mice. We therefore suggest that a negative feedback mechanism may exist in HBECs, where attenuation of membrane receptor expression may act as a measure to halt HDM-induced activation of Th2 inflammation.

Stratification of these observations based on genetic signals identified that this mechanism appears to be SNP dependent. Carriers of the asthma protective allele in Signal A (rs995514; CC) and Signal D (rs10192157; TC/TT) responded to HDM through elevations in soluble IL1RL1 protein. This observation ties in well with the hypothesis that increased levels of circulating soluble IL1RL1 protein may act as a decoy receptor for circulating IL-33, preventing activation of the transmembrane receptor and relates to our earlier observation that asthma risk allele carriers for *IL1RL1* variants present with lower soluble IL1RL1 protein. However, homozygote carriers of the asthma risk allele in Signal C (rs17027258; AA) also presented elevated levels of soluble IL1RL1 protein following HDM stimulation, suggesting potential roles of circulating soluble IL1RL1 that may induce asthma under certain conditions.

SNP stratification of *IL1RL1* expression at the mRNA level following HDM stimulation suggests that Signal B (rs4142132) is crucial in driving the attenuation of *IL1RL1* expression identified in the nongenotype selected experiments, as it was the only region to act as an eQTL in this regard. This effect appears to be driven by the eQTL effects at baseline, where sufficient *IL1RL1* levels for a negative feedback mechanism are only present in carriers of the protective allele.

Considering these findings in relation to *IL1RL1* inducible eQTLs, we present the signals at Signal B (rs4142132) and Signal C (rs72825929) as signals of particular importance for *IL1RL1* regulation both at baseline in cultured epithelial cells and in response to these allergic triggers. The Signal at position B (rs4142132) is associated with blood eosinophils, childhood asthma, atopy, type 2 inflammation, and asthma (20, 36), all relevant to allergic disease potentially driven by allergens such as HDM, whereas the Signal C (rs72825929) is associated with general allergic disease (4) and therefore also potentially has relevance to allergens such as HDM.

Interestingly, ENCODE data indicate that the effect on transcription factor motif binding for Signal C (rs72825929) is minimal, with an effect only on the Sox family of transcription factors, which have been linked with the normal development of the trachea and the lung (46–48). However, a greater number of motif changes disrupted by the remaining 3 polymorphisms, with significant overlap, can be observed. These include GATA binding motifs, where GATA2 is shown to regulate *IL1RL1* expression in mast cells and basophils (25) and has been shown to mediate the effects of HDM in the human lung (49). Of note also is that several of the eQTL SNPs resulted in changes in type 2 inflammatory transcription factors that are known to regulate *IL1RL1* (e.g., Gfi1). However, a large range of ubiquitously expressed transcription factors indicates that the effect on *IL1RL1* expression by different stimuli may be driven through different transcription factor binding motif interactions. While we present a comprehensive analysis of functional effects of *IL1RL1* region SNPs for *IL1RL1* gene expression and function, we acknowledge that functional effects on other genes ( eQTL effects on *IL18R1*, for example) may be of relevance, as well; however, these were beyond the focus of our current investigations to advance our understanding of the contribution of genetic variants to IL1RL1 biology in the context of asthma.

### Coding variants.

Finally, we examined the functional effects of the rs10192157 SNP that is in complete LD with several polymorphisms encoding 4 amino acid changes: Ala433/Gln501/Thr549/Leu551 being the asthma risk haplotype and Thr433/Arg501/Ile549/Ser551 being the protective haplotype. In agreement with other reports, we show that the asthma risk haplotype leads to enhanced NF-κB activity after IL-33–mediated *IL1RL1* signaling in a reductionist cell model (18, 23). It has been previously suggested that IL1RL1 may also affect TLR2 and TLR4 signaling (16), but we were unable to show supporting evidence for this in vitro. However, we do show that cells carrying the asthma risk IL1RL1 protein are more amenable to the antiinflammatory effects of anti-IL1RL1 and anti–IL-33, which has important therapeutic implications for potential stratified medicine approaches, especially in light of current pharmaceutical development of an IL-33/IL1RL1 antagonist for use in asthma.

### Conclusion.

This study has significantly advanced our understanding of both the phenotypic and the functional effects of polymorphisms in the *IL1RL1* locus in the context of asthma (Table 4). We have confirmed and extended genetic association to specific features of asthma, identifying 3 independent signals associated with blood eosinophil counts/asthma, lung function (FEV_1_), and severe asthma. Importantly, we have extended our understanding of Signal C (tagged by rs72825929), a previously reported signal for allergy (4) and self-reported asthma (50), associating it specifically to severe asthma. All 4 of the signals identified for functional analyses show effects on *IL1RL1* regulation, complementing and extending the literature, particularly by examining eQTLs in lung tissue and HBECs under different environments. However, some caveats in the data are observed — in particular, that asthma risk alleles at different signals have opposing effects on IL1RL1 expression, although this is supported by other studies and potentially highlights the complexity of this locus. Overall, these data suggest that asthma and asthma phenotype–related risk alleles, as part of distinct genetic signals at the *IL1RL1* locus, significantly affect *IL1RL1* mRNA and protein levels in a tissue- and isoform-specific way. Overall, our study therefore highlights the complexity of this susceptibility locus for asthma and identifies multiple signal-driven mechanisms that contribute to the genetic association signals, which, at least in part, explains why this locus represents one of the most reproducible association signals in asthma, to date.

## Methods

Supplemental Methods are available online with this article.

### Selection of genetic region and *IL1RL1* SNPs

#### Selection of region.

For the phenotypic analyses, we selected SNPs with a MAF ≥ 0.01 located in the genomic region 400 kb up- and downstream the *IL1RL1* gene (chr2: 102,527,961–103,368,497), which encompasses all of the previously described asthma signals, as well as several additional genes (Supplemental Figure 1). There were 3148 and 3048 SNPs with SNPTEST infoscore > 0.3 present in the GASP and DAG cohorts and 2760 SNPs with PLINK infoscore > 0.7 in Lifelines. Annotated SNP location and function was determined with the use of HaploReg v4.1 (51). All genetic data were annotated relative to assembly GRCh37/hg19. In the MAAS analyses, 2206 SNPs were available in the region for association testing.

#### Selection of SNPs for cell-based analysis.

SNPs of interest were selected using the following criteria: (a) significant association with asthma subtypes in our genetic association analysis (FDR < 0.05), (b) a MAF over 10% to facilitate subsequent in vitro analysis, and (c) independence based on r^2^ < 0.1 (in the 1000 genomes CEU population; ref. 17). SNP rs10192157 was also selected due to its nature as a functional SNP within *IL1RL1*. Levels of linkage disequilibrium were identified for SNPs at each stage of prioritization utilizing the online software LDlink (52, 53) (Supplemental Figures 9 and 10). From this, SNPs were prioritized based on LD (LD r^2^ < 0.1; Figure 2) and selected for further study (Table 1). A tagging SNP was chosen from each key haplotype block/signal of interest after the selection process ultimately gave 4 SNPs.

#### ENCODE.

We used data collected by ENCODE (54, 55) to identify potential functional significance of the associated SNPs within HaploReg v4.1 (51, 56). The data set was last accessed on the April 29, 2019, at 11:30 a.m.

### IL1RL1 TIR domain recombinant experiments

To examine differences in NF-κB signaling between TIR domain haplotypes after IL-33 stimulation in the presence and absence of anti–IL-33 or anti-IL1RL1, we used Kruskal-Wallis test followed by Bonferroni post hoc test. A *P* < 0.05 value was considered significant. In the TLR experiments, we tested 2 potential functional effects of the IL1RL1-b exon 11 haplotypes based on literature. First, we tested whether the 2 haplotypes IL1RL1-b showed a differential suppressive effect on TLR2 and TLR4 signaling, as previously reported for IL1RL1-b. IL1RL1-b exon 11 risk and protective haplotypes were overexpressed in HEK-Blue cells sensitive to either TLR2 stimulation with Pam3Cys or TLR4 stimulation with LPS at concentrations of 0, 0.1, 1.0, and 10 ng/mL.

### Statistics

We have included details of statistical tests and criteria used in each analysis in the online supplement; however, the following tests were used: (a) genotype-phenotype association testing (SNPTEST v2.5β or PLINK v1.90b6.7 with a FDR < 0.05 considered statistically significant), (b) resequencing in cases-controls association testing (Syzygy, *P* < 0.05 corrected for multiple testing [Bonferroni] was considered significant), (c) lung and bronchial biopsy eQTL (SNPtest v2.5β additive genetic model; for the 4 selected signals a *P* < 0.002 [Bonferroni correction] was considered significant), and (d) cultured cell eQTL, 2-tailed Mann-Whitney *U* or Kruskal-Wallis with Dunn’s correction for multiple testing; *P* < 0.05 was considered significant. IL1RL1 TIR domain recombinant experiments; Kruskal-Wallis with Dunn’s correction for multiple testing; *P* < 0.05 was considered significant.

### Study approval

The DAG and NORM cohort were approved by the Medical Ethics Committee of the UMCG. For Lifelines, all participants signed an informed consent form before they received an invitation for the physical examination. The Lifelines Cohort Study is conducted according to the principles of the Declaration of Helsinki and in accordance with the UMCG research code. The Lifelines study was approved by the medical ethical committee of the UMCG. The Lung eQTL study was approved by the ethics committees of the Institut universitaire de cardiologie et de pneumologie de Québec and the UBC-Providence Health Care Research Institute Ethics Board for Laval and UBC, respectively. The study protocol was consistent with the Research Code of the UMCG and Dutch national ethical and professional guidelines. In the AHBEC data set, brushes were collected under ethics REC 08/H0406/189 (University of Leicester) and REC 08/H0407/1 (University of Nottingham). All participants in this study provided informed consent. GASP is a multicenter study under ethics GM129901; however, it also includes samples collected under local ethics from Nottingham (recruited 1990-2015), Belfast (recruited 2008-2009), Birmingham (2005-2014), Manchester (recruited 2008-2014), Southampton (recruited 2003-2014), Glasgow (recruited 2002-2014), and Leicester (recruited 2004-2015). All studies had appropriate local ethics approval.

## Author contributions

Designing research studies was contributed by MAP, MEK, FND, MCN, GHK, and IS. Conducting experiments was contributed by MAP, MEK, FND, NSG, NS, JH, SB. Acquiring data was contributed by MAP, MEK, FND, NS, JH. Analyzing data was contributed by MAP, MEK, FND, MCN, NS, YB, CAB, CV, JH, A. Faiz, GHK, and IS. Providing samples and reagents was contributed by MAP, MEK, MO, APH, CKB, DS, SRJ, ZEKP, A. Faiz, TMM, DCN, YB, MVDB, A. Fogarty, SB, JMV, PDV, AS, LGH, AHM, RC, NCT, JWH, GAL, PHH, JH, RN, AS, JDB, MDT, DSP, IPH, LVW, MCN, CEB, GHK, and IS. Writing the manuscript was contributed by MAP, FND, MEK, MCN, GHK, and IS.

## Supplementary Material

Supplemental data

## Figures and Tables

**Table 4:**
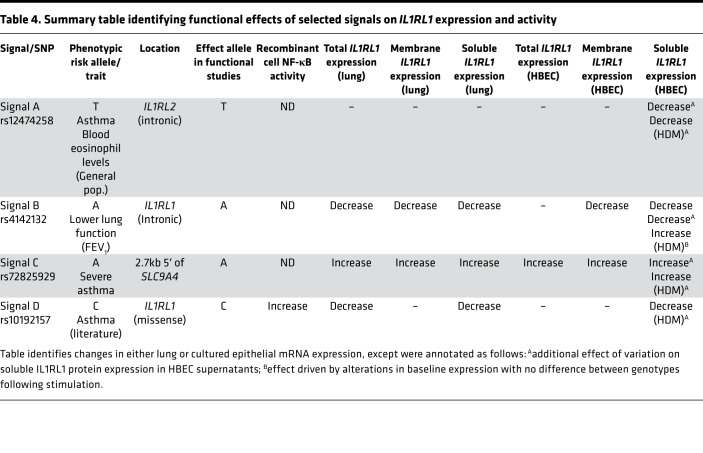
Summary table identifying functional effects of selected signals on *IL1RL1* expression and activity

**Table 1:**
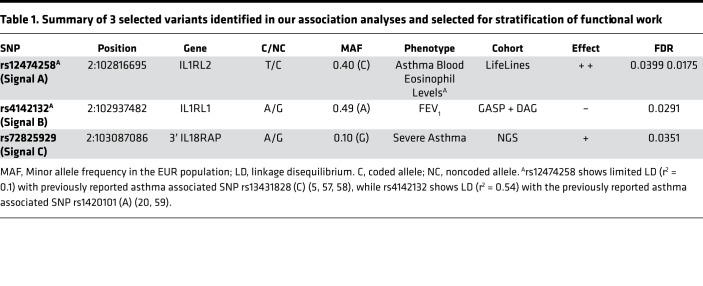
Summary of 3 selected variants identified in our association analyses and selected for stratification of functional work

**Table 2:**
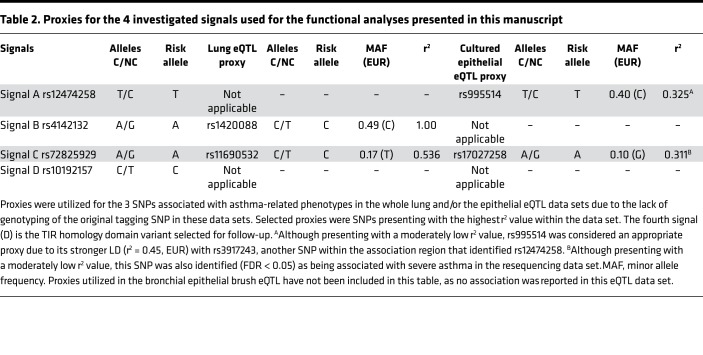
Proxies for the 4 investigated signals used for the functional analyses presented in this manuscript

**Table 3:**
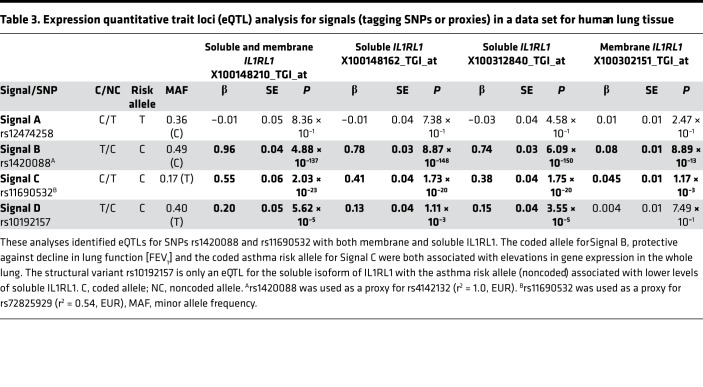
Expression quantitative trait loci (eQTL) analysis for signals (tagging SNPs or proxies) in a data set for human lung tissue

## Version 1 04/23/2020

Electronic publication
