# Supplementary material for: Phenotypic and functional translation of IL1RL1 locus polymorphisms in lung tissue and asthmatic airway epithelium
Source: JCI Insight. 2020 Apr 23;5(8):e132446. doi: 10.1172/jci.insight.132446 (PMC7205441; doi:10.1172/jci.insight.132446)
Supplement: Supplemental data [file jciinsight-5-132446-s011.pdf]

Supplemental material

Phenotypic and functional translation of *IL1RL1* locus polymorphisms in lung tissue and airway epithelium in asthma

~~Michael A. Portelli[1*], Nicole F. Dijk[2a*], Maria E. Ketelaar[1,2ab*], Nick Shrine[3], Jenny Hankinson[4], Sangita Bhaker[1], Néomi E. Grotenboer[2ab], Ma'en Obeidat[5], Amanda P. Henry[1], Charlotte K. Billington[1], Dominick Shaw[1], Simon R. Johnson[1], Zara E.K. Pogson[6], Andrew Fogarty[6], Tricia M. McKeever[6], David C. Nickle[7], Yohan Bossé[8], Maarten van den Berge[2c], Alen Faiz[2c], Sharon Brouwer[2b], Judith M. Vonk[2d], Paul de Vos[2b], Amisha Singapuri[9], Liam Heaney[10], Adel H. Mansur[11], Rekha Chaudhuri[12], Neil C. Thomson[12], John W. Holloway[13], Gabrielle A. Lockett[13], Peter H. Howarth[13], Robert Niven[4], Angela Simpson[4], John D. Blakey[14], Martin D. Tobin[3, 15], Dirkje S. Postma[2c], Ian P. Hall[1], Louise Wain[3, 15], Martijn C. Nawijn[2b], Christopher E. Brightling[8, 15], Gerard H. Koppelman[2a#], Ian Sayers[1#]~~

~~**shared first authors; #shared senior authors*~~

~~*[1]Division of Respiratory Medicine, National Institute for Health Research, Nottingham Biomedical Research Centre, University of Nottingham, Nottingham, UK.*~~
~~*[2]University of Groningen, University Medical Center Groningen, Groningen Research Institute for Asthma and COPD, Groningen, The Netherlands*~~
~~*[a]University of Groningen, University Medical Center Groningen, Department of Pediatric Pulmonology and Pediatric Allergology, Beatrix Children's Hospital, Groningen, the Netherlands*~~
~~*[b]University of Groningen, University Medical Center Groningen, Department of Pathology and Medical Biology, Groningen, The Netherlands*~~
~~*[c]University of Groningen, University Medical Center Groningen, Department of Pulmonary Diseases, Groningen, The Netherlands.*~~
~~*[d]University of Groningen, University Medical Center Groningen, Department of Epidemiology, Groningen, The Netherlands*~~
~~*[3]Department of Health Sciences, University of Leicester, Leicester, UK*~~
~~*[4]Manchester Academic Health Science Centre, University of Manchester, Manchester, UK*~~
~~*[5]The University of British Columbia Center for Heart Lung Innovation, St Paul's Hospital Vancouver, Vancouver, BC, Canada.*~~
~~*[6]Division of Epidemiology and Public Health, University of Nottingham, Nottingham, UK.*~~
~~*[7]Departments of Genetics and Pharmacogenomics, Merck Research Laboratories, Boston, Massachusetts, USA*~~
~~*[8]Institut universitaire de cardiologie et de pneumologie de Québec, Department of Molecular Medicine, Laval University, Québec, Canada*~~
~~*[9] Respiratory sciences, University of Leicester, Glenfield Hospital, Leicester, UK*~~
~~*[10]Centre for Infection and Immunity, Queen's University of Belfast, Belfast, UK*~~
~~*[11]Respiratory Medicine, Birmingham Heartlands Hospital and University of Birmingham, Birmingham, UK*~~
~~*[12]Institute of Infection, Immunity and Inflammation, University of Glasgow, Glasgow, UK*~~
~~*[13]Human Development & Health & Clinical and Experimental Sciences, Faculty of Medicine and National Institute of Health Biomedical Research Centre, University of Southampton, Southampton, UK.*~~
~~*[14]Department of Clinical Sciences, Liverpool School of Tropical Medicine, Liverpool, UK*~~

[15] *National Institute for Health Research, Leicester Respiratory Biomedical Research Centre, University of Leicester, Leicester, UK.*

Supplemental Results

Demographics

Five cohorts were included in this study: Two clinical cohorts (asthma cases only), the Genetics of Severe Asthma Phenotypes (GASP) (1), and Dutch Asthma Genetics (DAG) (2); two population based cohorts (providing asthma cases and controls), the Manchester Asthma and Allergy Study (MAAS) (3) and the Lifelines Cohort Study (4) and the Nottingham Gedling Cohort (5) (providing controls only). General characteristics of cohorts are represented in Supplemental Tables 1-3. The GASP cohort provided 2,536 cases where genotypes were available for analyses. This cohort had a mean subject age of 47.8 years, a mean age of asthma onset of 23.2 years (46.2% being diagnosed with asthma before the age of 16 years) and 46.8% atopic prevalence. The DAG cohort provided 909 cases with genetic data, with a mean age of 34.8 years, mean age of asthma onset of 10.1 years (57.2% being diagnosed with asthma before the age of 16 years) and 63.7% atopic prevalence. The MAAS included 919 children with available genotypes, presenting with a mean age of 8 years, (23.9% being diagnosed with asthma) and 32.9% atopic prevalence. The Lifelines cohort included 1066 asthma cases (mean age 46.2 years) and an additional 6,863 controls (mean age 49.2 years); no data was available for atopic status, total IgE or onset of asthma. The Nottingham Gedling cohort provided 200 non-asthmatic controls (mean age 57).

Association of *IL1RL1* variants with characteristics of asthma

In our Stage 1 analyses (Figure 1) we identified 461 SNPs associated with one or more of the asthma related traits across the association analyses using the initial FDR<0.05 screen. In particular, 130 SNPs showed association with asthma diagnosis and 316 with blood eosinophil counts in the general population in the LifeLines cohort (Supplemental Tables 8 & 9). This consisted of two signals (r^2>0.1) each contained within its own LD block, where both signals were common to both asthma diagnosis and eosinophil levels in a general population (rs12474258 (*IL1RL2* intronic) (T): Asthma; OR=1.20 (SE 0.05), FDR=0.049, Eos Counts; Beta=0.027 (SE 0.008), FDR=0.017; rs72823628 (*IL1RL1*

intronic) (G): Asthma OR=1.38 (SE 0.09), FDR=0.049, Eos Counts; Beta=0.013 (SE 0.013), FDR=0.046). Significant overlap was observed between asthma associated SNPs and SNPs associated with eosinophil levels in a general population (please refers to Supplemental Table 8). Similarly, 4 SNPs showed association with atopy in a meta-analysis of the GASP & DAG cohorts (Sentinel SNP: rs2041747 (*IL1R1*) (A) meta-analysis; Beta =-0.63 (SE 0.19), *P*=7.81E-04). This consisted of a single signal (r^2>0.1) (Supplemental Table 3). However this signal did not achieve later criteria of MAF≥0.1 and was therefore not taken forward. An additional two association signal consisting of a single SNP each at FDR<0.05, was also observed for lung function (FEV_1) in the meta-analysis (Sentinel SNP: rs4142132 (*IL1RL1* intronic) (A); Beta=-0.07 (SE 0.02), FDR=0.029; Sentinel SNP: rs113238379 (*SLC9A2* intronic) (A); Beta=-0.21 (SE 0.07), FDR=0.049) (Supplemental Table 7). However the signal tagged by rs113238379 did not achieve later criteria of MAF≥0.1 and was therefore not taken forward.

Overall this identified 2 independent signals across the asthma-subtypes consisting of two or more SNPs in LD. Association testing in the childhood cohort (MAAS) for lung function and atopy phenotypes evaluated 2,206 SNPs for association and identified no signals meeting initial criteria of an FDR less than 5%.

To identify novel variants, including potentially rare variants, we undertook two sequencing approaches. By resequencing the exons and additional regions of *IL1RL1* in 94 asthma patients using Sanger sequencing we identified 56 variants (Supplemental Table 3) with 14 resulting in amino acid changes within *IL1RL1*. Of these 56 SNPs, 40 were common and 16 rare (Minor Allele Frequency [MAF]<0.05) in our adult asthma population (N=94) (see Supplemental Table 3). No novel SNPs were identified. Using a complimentary approach that also integrates intronic and intergenic regions, in parallel we enriched the entire chromosome 2q12 locus for region based sequencing using DNA from 200 severe asthma subjects and 2000 non-asthmatic, non-atopic control subjects. These analyses identified 4,107 variants spanning the region (Supplemental Table 1); including 51 variants (37 non-synonymous, 14 synonymous) in the coding regions of *IL18R1, IL18RAP, IL1R1, IL1RL1, IL1RL2* & *SLC9A4* with nine coding region variants in the *IL1RL1* gene (Supplemental Table 1). Additional

variation including structural variation e.g. insertion/deletions spanning the region were also identified (Supplemental Table 1).

Using region based sequencing data generated in the case/control cohort we completed an association analysis for severe asthma as an outcome using sequencing allele counts. Using the same initial screening of association (FDR<0.05) we identified 8 variants meeting criteria (Supplemental Table 2). This included a novel SNP at location chr2:103141056 (C|T) with a population frequency of 0.0271. The most significant variant, a previously unreported signal; rs35404747 (*IL1R1*), generates an insertion (C/CCTT), case MAF: 0.0009; control MAF: 0.1093, FDR=8.21E-08 (Supplemental Table 2). However, the only variant to achieve later criteria of (MAF≥0.1) was rs72825929 (5` *SLC9A4*), (A), case MAF: 0.08; control MAF: 0.18, FDR=0.035 (Supplemental Table 2).

IL1RL1 SNPs were not identified as eQTLs in airway wall biopsies in healthy subjects

Next, we extended eQTL analysis to bronchial brushings and biopsies to test whether the selected signals are associated with *IL1RL1* expression in airway wall. Examination of the four priority signals for effects on *IL1RL1* expression in bronchial brushings (n=72) as well as in bronchial biopsies (n=77) of healthy volunteers, however, did not identify any eQTLs (Supplemental Table 14).

Supplemental Methods

Cohorts

Genetics of Asthma Severity and Phenotypes (GASP) cohort

Asthmatic individuals aged 16-60 were recruited from across the United Kingdom as part of an Asthma UK initiative to generate a cohort that is enriched for patients with British Thoracic Society Step 3 and above (moderate-severe asthma) (6). Subjects had extensive clinical characterisations (Supplemental Table 10). Asthma was defined as a doctor's diagnosis of asthma through the presence of symptoms and medical treatment, while age of onset of asthma was determined through patient records. Asthma

related clinical traits used in the current study focussed to lung function (FEV_1, FEV_1/FVC), atopic status (positive skin prick test), Blood Eosinophil Count ($x10^9/L$) and Blood IgE levels (KU/L). Lung function tests for FEV_1 and FVC were carried out through spirometry using a Vitalograph Medisoft BB5500 Whole Body Plethysmography System. Total peripheral blood eosinophil levels were calculated using a counting chamber while total Immunoglobulin E (IgE) levels were measured by ImmunoCAP™. Finally, atopy was defined as a positive response to a skin prick test (SPT) to any allergen from a panel of 4-24 allergens.

Dutch Asthma GWAS (DAG) cohort

The DAG cohort consists of 469 trios (patient – spouse – child) ascertained through a proband with asthma, combined with an additional case-control study of 452 asthmatics and 511 controls (2). Of these, we selected 909 asthma patients who underwent the same, standardized, comprehensive evaluation for asthma at Beatrixoord Hospital, Haren, The Netherlands between 1962-2000. Asthma was defined as a doctor's diagnosis of asthma, asthma symptoms, and presence of bronchial hyper-responsiveness (BHR). FEV_1 was measured using a water-sealed spirometer (Lode Spirograph type DL, Lode b.v., Groningen, The Netherlands). Total peripheral blood eosinophils were counted in a counting chamber and IgE levels were measured in serum by an enzyme-linked fluorescence assay (Mini Vidas, Biomerieux Inc., Marcy, France). In subjects older than 12 years, intracutaneous tests with 16 common aeroallergens were performed. In children younger than 12 years, a skin prick test was performed with 10 allergens. Subjects with a positive response to one or more intracutaneous or skin prick tests were considered to be atopic. Age of asthma onset was based on data from medical records and questionnaires, indicating the start of asthma symptoms (Supplemental Table 10). A representative subset (n=95) of the DAG cohort was taken for resequencing the *IL1RL1* region, and consisted of asthma patients of moderate severity, as shown in Supplemental Table 11.

Lifelines Cohort

Lifelines is a multi-disciplinary prospective population-based cohort study examining in a unique three-generation design the health and health-related behaviours of 167,729 persons living in the North of The Netherlands. It employs a broad range of investigative procedures in assessing the biomedical, socio-demographic, behavioural, physical and psychological factors which contribute to the health and disease of the general population, with a special focus on multi-morbidity and complex genetics. The cohort profile of the Lifelines study has been extensively described previously (4) (see Table 1 for sub-set used in this analysis). Participant baseline visit took place between December 2006 and December 2013. All general practitioners in the three northern provinces of the Netherlands were asked to invite their registered patients aged 25–49 years. All persons who consented to participate were asked to provide contact details to invite their family members (i.e., partner, parents and children), resulting in a three-generation study. Baseline data were collected from 167,729 participants, aged from 6 months to 93 years. Collected data include physical examinations, DNA, blood and urine samples, and comprehensive questionnaires on history of diseases, quality of life, lifestyle, individual socioeconomic status, work, psychosocial characteristics and medication use. Follow-up is planned for at least 30 years, with questionnaires administered every 1.5 years and a physical examination scheduled every 5 years. At current, a subset of the adult participants have genomewide genotype information available (n=13,395), numbers still increasing with each novel release of Lifelines every couple of years.

Lifelines is a facility that is open to all researchers. An overview of the available data and application procedure is presented online, accessible via www.lifeline[illegible].

Manchester Asthma and Allergy Study (MAAS) cohort

MAAS is a birth cohort study which follows the development of both asthma and other atopic disorders in a population based birth cohort. Subjects were recruited in the maternity catchment area of Wythenshawe and Stepping Hill Hospitals, comprising 50 square miles of South Manchester and Cheshire, United Kingdom (Supplemental Table 13). Clinical follow up took place at age 8 years. FEV_1 and FVC were measured according to the American Thoracic Society guidelines (7) using a

pneumotachograph based spirometer using incentive animation software (Jaeger, Germany). The test was repeated at intervals of 30 seconds until 3 technically acceptable traces were obtained and the highest FEV_1 was recorded. Atopy was defined as a positive skin prick test (mean weal diameter 3mm greater than negative control) to at least one of the allergens tested (Mite, cat, dog, grass pollen, milk, egg, mixed moulds, tree pollen, peanut). Asthma was defined as parental report to the question "has your child ever suffered from asthma?"

Lung Tissue Cohort

Non-tumor containing lung tissue was collected from patients who underwent lung resection surgery at three participating sites: Laval University (Quebec, Canada), University of British Columbia (Vancouver, Canada) and University Medical Center Groningen (Groningen, The Netherlands). Cohort characteristics have been described previously (8).

Normal Values of Inflammatory Variables From Healthy Subjects (NORM) cohort

77 respiratory healthy subjects were derived from the Normal Values of Inflammatory Variables From Healthy Subjects (NORM) study (NCT00848406 (9, 10)). Current smokers and never smokers older than 18 years were recruited into this study. Subjects were considered respiratory healthy if they had no respiratory symptoms, no history of respiratory disease and normal pulmonary function. Normal pulmonary function was defined as a post bronchodilator FEV_1/FVC higher than lower limit of normal, absence of AHR to methacholine (PC20 <16mg/mL) and absence of reversibility (FEV_1 %predicted to Salbutamol < 10%). Subjects were excluded if they used inhaled or oral corticosteroids within the last 5 years, or for a period of 5 years or longer.

The Asthma Human Bronchial Epithelial Cell (AHBEC) cohort

The asthma human bronchial epithelial cell (AHBEC) cohort consists of primary bronchial epithelial cells from 33 individuals with a doctor's diagnosis of asthma recruited from Nottingham and Leicester. Cells were obtained through endobronchial biopsies and brushes carried out as per standard procedure (11, 12). These cells were used for all primary human cell culture, as described. Clinical characterization of these patients included lung function tests for FEV_1 and FVC. Total peripheral blood eosinophil levels were calculated using a counting chamber (Supplemental Table 15).

Functional characterisation of *IL1RL1* variants in primary bronchial epithelial cells

Cell culture and collection of RNA and protein lysate.

Passage 2/3 HBECs obtained from bronchial brushes and biopsies from the AHBEC cohort as previously described (13), were cultured on PureCol Type-I Bovine collagen (Advanced BioMatrix, 5005-B) in fresh growth factor-supplemented medium (BEGM, Lonza). BEGM was changed to basal medium 24 hours prior to stimulation with either HDM (50µg/ml) (Greer XPB70D3A25 (Lot: 23187)), human recombinant IL-33 (50ng/ml) (Abnova, P3638) or PBS as a vehicle control. For stimulation with RV16 (Public Health England), BEGM was changed to infection medium (BEGM-I), i.e. BEGM lacking Bovine Pituitary Extract, 24 hours prior to infection with a virus MOI of 1. Cells were infected for a period of 1 hour, following which they were washed three times with sterile PBS and fresh BEGM-I was added. Cells were then incubated for 24 hours. Protein and RNA lysates were collected as previously described (14).

Quantitative PCR

HBEC complimentary DNA (cDNA) was synthesised from 1µg RNA using Superscript II (Invitrogen, UK) and random hexamer primers according to the manufacturer's instructions. TaqMan® Quantitative PCR (qPCR) was then utilised to quantify mRNA levels of *IL1RL1* and was performed and analysed as previously described (14).

Genotyping of primary bronchial epithelial cells

DNA was extracted using the Qiagen QIAamp® DNA Mini and Blood Mini Kit according to the manufacturer's instructions. SNP Genotyping was then carried out using TaqMan® Pre-designed assays.

Quantification of soluble IL1RL1 protein levels.

Soluble IL1RL1 in cellular supernatants was measured using Luminex assays (supplied by R&D, product code LXSAHM) according to the manufacturer's recommendations using a custom Magnetic Luminex Screening Assay with a Human Premixed Multi-Analyte Kit (R&D systems). Each experimental supernatant was assayed in duplicate.

Functional characterisation of *IL1RL1* coding region variants using recombinant expression

Preparation of IL1RL1 coding region transfection plasmids.

The open reading frame of membrane *IL1RL1* containing the exon 11 asthma protective/risk haplotypes for the TIR domain haplotype was amplified from mRNA to generate two expression cassettes containing either; Ala433-Glu501-Thr549-Leu551 or Thr433-Arg501-Ile549-Ser551 haplotypes. Primers including a consensus Kozak sequence and restriction enzyme sites: 5'primer: 5'-ACTTGCTAGCGCCACCATGGGGTTTTGGATC-3' and 3' primer: 5'-ACTTGCGGCCGCCTATTGCTTCTGG GCAGCC -3'. The PCR products were cloned into the pGEM®-T Vector System II (Promega A1380), subcloned (NheI and NotI) into pCDH-CMV-MCS-EF1-copGFP (SBI) and sequence verified.

IL1RL1-HEK Reporter cells system.

HEK293 cells genetically modified to contain an NF-KB Secreted Alkaline Phosphatase Reporter (NovusBio NBP2-26260), were cultured in DMEM containing 4.5 g/L -glucose and 4 mM L-glutamine (Sigma D5796) supplemented with 10% FBS, 1 mM sodium pyruvate, 100 units/ml Penicillin, 100µg/ml Streptomycin and 500µg/ml G418. Cells were transfected with the *IL1RL1* plasmids in a 96-

well plate format using the TransIT-LT1 lipid transfection reagent (Mirus Bio, MIR2300) as per manufacturer's instructions. Empty vector (pCDH-CMV-MCS-EF1-copGFP) was used as a transfection control and adjustments were made to correct for differences in plasmid size. Following a 24 hour period, cells were incubated for 1 hour with either 10µg/ml of either an anti-IL-33 (Invivogen, mabg-hIL-33-3) or anti-IL1RL1 antibody (R&D, MAB523) followed by stimulation with a range of concentrations of human recombinant IL-33 (ABNOVA, P3638). NF-KB -activated SEAP release was measured 24 hours following IL-33 stimulation, using a commercial SEAP Reporter Assay Kit (Invivogen, rep-sap). 10µl of cellular supernatants were used and activity measured as per manufacturer's instructions. For the TLR experiments, an analogous system was used, however HEK293-NF-KB cells containing recombinant TLR2 or TLR4 prior to IL1RL1 transfection were used and these experiments used an earlier version of the IL1RL1 TIR plasmids (in in pcDNA3.1).

Western blots

HEK293T cells stably transfected with the protective or the risk haplotype of IL1RL1 (in pcDNA3.1) were seeded into 6 well plates (10^6 cells / well) in DMEM (10%FCS), cultured for 24h, followed by serum starvation (0% FCS/DMEM) overnight. Cells were stimulated after serum starvation with IL-33 at 30 ng/ml for 0/10/20/30 minutes in DMEM (0%FCS), followed by aspiration of the media and lysis of the cells using Laemmli buffer (200 µL per well), scraping of the well and collection of the lysate. Lysates were immediately boiled for 10 minutes and stored at -80. Lysates were analyzed using 10% PAA gel (home made by standard TRIS/SDS/Acrylamide/APS/TEMED) followed by transfer to nitrocellulose using methanol-containing blotting buffer (200 Amp, 2 hours). Blocking of the nitrocellulose is in 5% ELK/TBS, washing in 1x TBST prior to probing with total or phospho-specific ERK1/2 antibody (respectively mAb#4695, clone 137F5; and mAb#4370; clone D13.14.4E; CellSignallingTechnology).

Genotyping

GASP

Participants in the GASP cohort were genotyped using two platforms, 744 subjects initially genotyped using the Affymetrix Axiom® UK BiLEVE array and 2172 subjects genotyped subsequently using the

related Affymetrix Axiom® UK Biobank array. In each genotyping batch samples were excluded if: (i) their genetically inferred gender did not match their reported gender; (ii) they had outlying heterozygosity within the batch (outside either 2 or 3 standard deviations from the mean depending on batch); (iii) they had a call rate < 95% across genotyped variants; (iv) they were cryptically related to another sample, in which case 1 sample of the pair was removed; (v) the sample shows significant deviation from European ancestry as determined by a plot of the first two principal components. Failed genotyping quality control excluded 380 subjects. Therefore, following exclusion of monomorphic SNPs and SNPs only found on a single array, 692,060 SNPs were available for 2,536 subjects.

DAG

Participants in the DAG cohort were genotyped on two platforms, the Illumina 317 Chip and the Illumina 370 Duo Chip (Illumina, San Diego, CA). Quality control (QC) was performed per chip with exclusion of individuals with a missing genotype call rate >0.01, related individuals (identity by descent sharing (IBS) >0.125) and non-Caucasian subjects, as assessed by principal components analysis performed with EIGENSTRAT (15). SNPs were excluded with a missing genotype rate >0.01, a Hardy-Weinberg equilibrium P-value $<10^{-7}$ and a MAF <0.01. Markers with Mendelian errors in phase I were excluded from analysis. Following quality control, the chips were merged and SNPs not available in both cohorts were excluded from the dataset. A total of 294,775 SNPs remained. Imputation was performed using IMPUTE 2.0 against the reference data set of the CEU panel from the 1000 Genomes project (version March 2012) (16). A subset of this cohort (n=94) was selected for targeted resequencing of the *IL1RL1* gene. We developed 23 primer pairs with primer 3 software spanning the distal promoter region, exon 1a, intron 1a, proximal promotor, exon 1b, intron 1b, exon 2, exon 3, exon 4, exon 5, intron 5, exon 5E, intron 5E, exon 6, exon 7, intron 7, exon 8, intron 8, exon 9, exon 10, intron 10 and exon 11. PCR products were Sanger-sequenced with Applied Biosystems 3730x1 DNA analyser. DNA variants were analysed using Mutation Surveyor Software.

Lifelines Cohort

Participants of the Lifelines cohort were genotyped on the HumanCytoSNP-12 BeadChip (Illumina). Quality control before imputation was performed using ImputationTool2, excluding SNPs with a call-rate <95%, with a HWE-P value <0.001, MAF <0.01%. Samples were excluded in case of ambiguous sex (genetic mismatch with reported sex), of non-Caucasian origin (based on self-report, IBS and population stratification using EIGENSTRAT (57)), and in case a pair of samples was discovered as first degree relatives using genetic cryptic relatedness, the sample with the best genotype quality was included only. Imputation was performed through Beagle 3.1.0 against the CEU panel from the 1000 genomes project (version March 2012) (58).

MAAS

For the MAAS cohort, DNA was extracted from a total of 1025 individuals, who were genotyped using the Illumina Human610-Quad array providing 2206 SNPs for analyses post QC in the chromosome 2 region.

Lung Tissue

In the Lung eQTL dataset, DNA samples were genotyped with Illumina Human1M-Duo BeadChip arrays, and gene expression profiles were obtained using a custom Rosetta/Merck Human RSTA Custom Affymetrix 2.0 microarray. Gene expression data are available on the Gene Expression Omnibus accession number GSE23546 and platform GPL10379. Genotyping was completed using the Human 1M-Duo BeadChip array (Illumina Inc, San Diego, USA) which was imputed against the 1000G phase 1 reference panel (EUR) using IMPUTE2. Imputed SNP data were available for 1,095 of the 1,111 subjects. The final dataset included n=1088 subjects.

Bronchial brushes and biopsies

In the NORM study, genotypes have been determined using DNA from peripheral blood mononuclear cells (PBMCs) or oral swabs using two platforms: the Human CytoSNP 12 and OmniExpress Exome

genotyping arrays (both Illumina Inc, San Diego, USA). Imputation was completed using the Haplotype Reference Consortium Release 1.1 using the Michigan imputation server. Imputed SNP data was available for n=72 subjects.

SureSelect bait design for region based next generation sequencing

The SureSelect method requires the design of 120 base pair oligonucleotide 'baits' in order to carry out sample target enrichment. Bait design was carried out using the SureSelect™ e-array design software available at https://earray.chem.agilent.com/earray as directed by the manufacturer.

Next-Generation DNA Sequencing (NGS).

DNA from 200 severe asthma cases (BTS 4, 5) from GASP and 200 non-asthmatic, non-atopic, non-wheeze controls from the Nottingham Gedling cohort (5), were selected for resequencing. Subjects were matched for age and gender (Supplemental Table 12). Next-generation Illumina sequencing of the 2q12.1 region was outsourced to Source Bioscience (Nottingham, UK) and was carried out using the SureSelect enrichment approach. The chromosome 2 locus previously associated with asthma (17, 18) [GRCh37.p9] was the focus and 120 base pair paired-end long read oligonucleotides (baits) were designed using the SureSelect™ e-array design software. Bait tiling (X5) was used across the region, presenting with a capture size range of 500Kb to 1.5Mb. The initial target region was 470,788bp; using 13,606 baits achieved 79.58% coverage of this region. Samples were pooled for sequencing (3 pools for cases and 3 pools for controls; each set of 3 pools consisting of 66, 66 & 68 subjects). Next-generation sequencing was carried out on these six samples on two separate lanes, one for cases and the other for controls, using the Illumina HiSeq2000™ systems pipeline (San Diego, USA). Sequencing used a paired end design using 100bp reads.

Following alignment to the NCBI GRCh37 genome template with Burrows-Wheeler Aligner (19), and removal of duplicates, 14.1 million reads mapped to the 2q12.1 region, giving a mean depth of 46× per sample. Realignment around insertion deletions and recalibration of quality scores was carried out using the freeware software programme GATK (20), while novel variant detection was carried out using the freeware software program Syzgy® (21). In SNVer, default read mapping quality [20] and base calling

quality [17] thresholds were used for variant calling, while in Syzgy® variants were detected using a quality threshold of 10 (accuracy 90%) and compared to the NCBI SNP database (dbSNP) to determine known variation in the region. Syzygy® flags variant quality (Supplementary Table 6) as "moderate", "poor", "very high" or "in cluster", the latter indicating that there is another variant within 2bp and hence a quality cannot be calculated.

Variant quality was obtained by both SNVer and Syzygy® (Supplementary Table 1). SNVer uses the fisher exact threshold to remove potential false positives due to allele imbalance issues, tagging variants as 'TRUE' or 'FALSE'. Syzygy® flags variant quality as "moderate", "poor", "very high" or "in cluster", the latter indicating that there is another variant within 2bp and hence a quality cannot be calculated.' We selected variants based on positive association with asthma that were flagged as high quality by Syzygy® and 'TRUE' by SNVer.

Bronchial brushes and biopsies.

In the NORM study bronchial brushings and biopsies were taken from the segmental divisions of the main bronchi in separate bronchoscopy sessions. Bronchial brushes were collected using a Cellebrity brush (Boston Scientific, Massachusetts, USA) and analyzed for genome wide gene expression using a Human Genome ST v1.0 micro-array. Bronchial biopsies were processed for RNA-sequencing. The methods for RNA extraction, labeling, processing and expression quantification are described in the Supplementary methods.

Normal Values of Inflammatory Variables From Healthy Subjects (NORM) study

RNA extraction, Sample preparation and gene expression quantification

Bronchial brushes and biopsies were taken from segmental divisions of the main bronchi in separate bronchoscopy sessions. Brushes were analyzed for RNA expression using micro-array, biopsies were processed for RNAsequencing.

Brushes: bronchial brushes were collected using a Cellebrity brush (Boston Scientific, Massachusetts, USA). After collection, they were immediately placed in RNAprotect Cell Reagent (Qiagen, Venlo,

The Netherlands) and stored at −80°C. Total cellular RNA was isolated using the Qiagen total RNA isolation kit. Samples were then randomized, labeled, and run on Affymetrix Human Gene chip ST1.0 arrays as described previously according to manufacturer's instructions (22). Microarray analyses were performed using R (v3.3.2) limma package and normalization was conducted in a single batch using Robust Multi-array Average.

Biopsies: Biopsies frozen in Tissue-Tek (VWR, Radnor, PA) at -80°C were thawed at room temperature and cut from the blocks when they were semi-solid. Total RNA was extracted using AllPrep DNA/RNA Mini kit (Qiagen, Venlo, the Netherlands). Samples were lysed in 600µl RLT-plus buffer using an IKA Ultra Turrax T10 Homogenizer, and RNA was purified according to the manufacturer's instructions. RNA samples were dissolved in 30µl RNAse free water. Concentrations and quality of RNA were assessed using a Nanodrop-1000 and run on a Labchip GX (PerkinElmer, Waltham, MA). RNA samples were further processed using the TruSeq Stranded Total RNA Sample Preparation Kit (Illumina, San Diego, CA), using an automated procedure in a Caliper Sciclone NGS Workstation (PerkinElmer, Waltham, MA). In this procedure, all cytoplasmic and mitochondria rRNA was removed (RiboZero Gold kit). The obtained cDNA fragment libraries were loaded in pools of multiple samples onto an Illumina HiSeq2500 sequencer using default parameters for paired-end sequencing (2 × 100 bp).

The trimmed FastQ files where aligned to build b37 of the human reference genome using HISAT (version 0.1.5) allowing for 2 mismatches (23). Before gene quantification SAMtools (version 1.2) was used to sort the aligned reads (24). The gene level quantification was performed by HTSeq (version 0.6.1p1) using Ensembl version 75 as gene annotation database (25).

Quality control (QC) metrics were calculated for the raw sequencing data, using the FastQC tool (version 0.11.3) (26). QC metrics were calculated for the aligned reads using Picard-tools (version 1.130) (http://picard.sourceforge.net) CollectRnaSeqMetrics, MarkDuplicates, CollectInsertSize-Metrics and SAMtools flagstat. In addition, we checked for concordance between sexlinked (*XIST* and Y-chromosomal genes) gene expression and reported sex. All samples were concordant.

Supplemental Figures

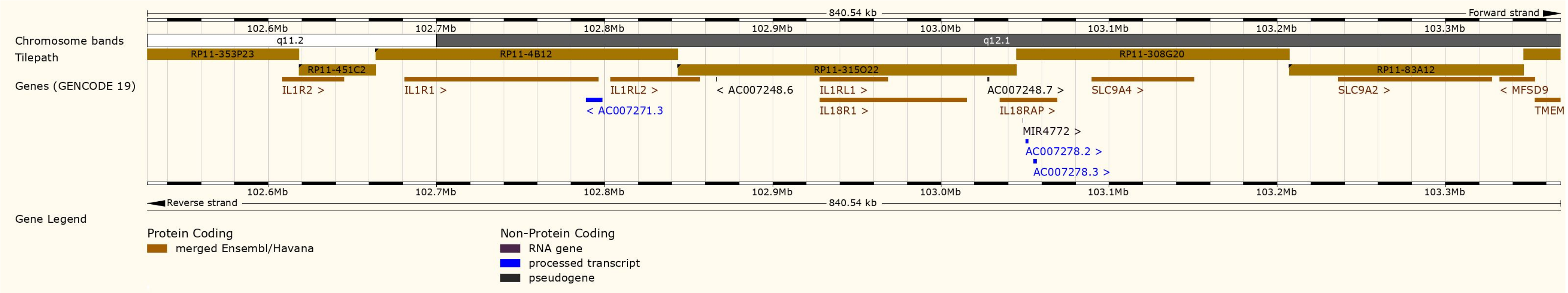

Supplemental Figure 1. Overview of genomic region used for phenotypic association testing. The region encompasses 400kb up- and downstream of *IL1RL1* (chr2: 102,527,961-103,368,497). Figure was made with the use of Ensembl Data.[21]

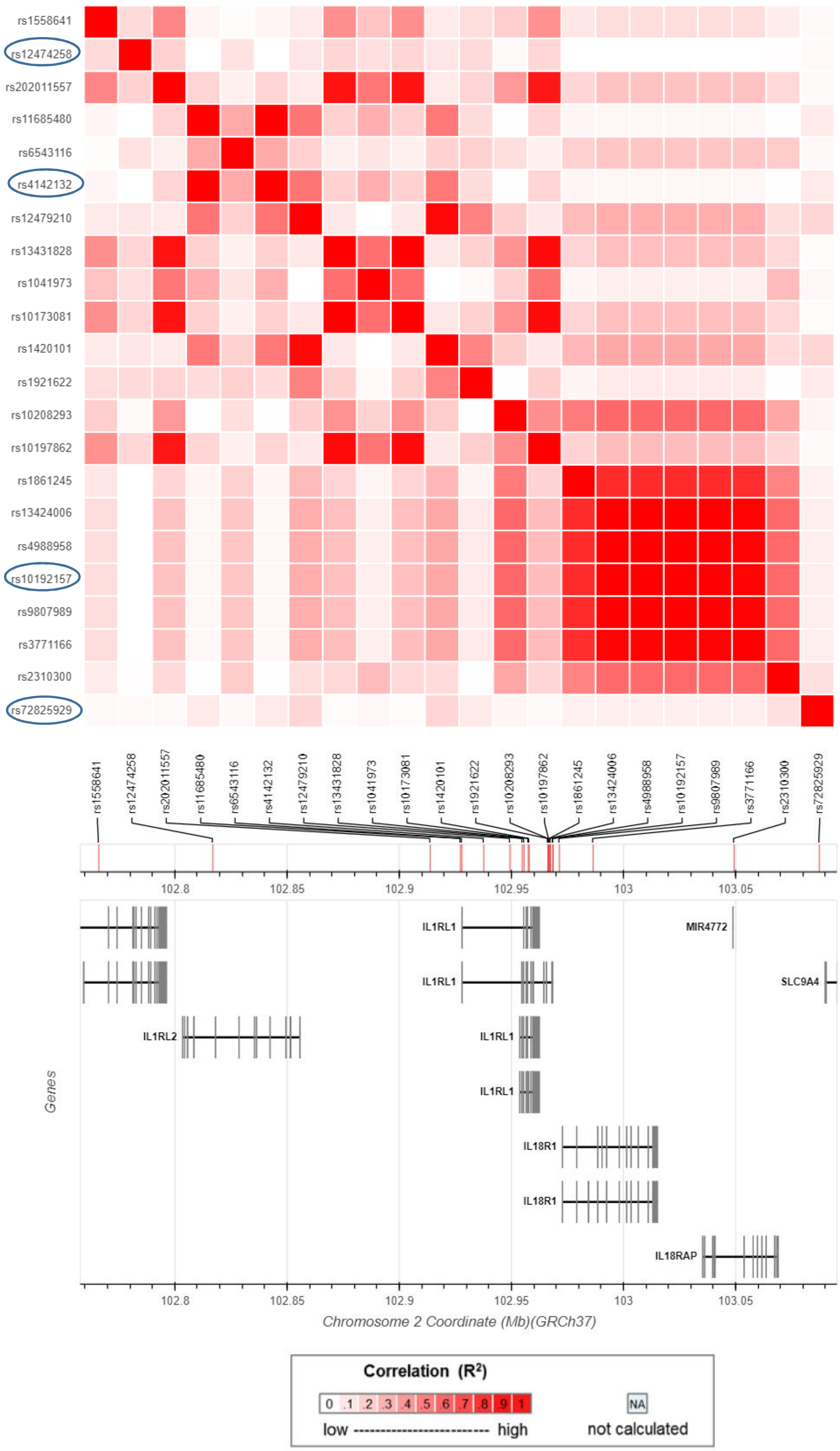

Supplemental Figure 2. ***Linkage disequilibrium map of the three variants identified in Stages 1-3 and the tagging SNP for the TIR domain haplotype, with previously published asthma associated SNPs in the chromosome 2 region.*** *SNPs discussed in this manuscript are highlighted in blue. Figure identifies the level of LD between signals identified based on r^2 values. Image generated using the EUR*

population of the Phase I cohort of the 1000 genomes study via the LDmatrix tab of the online software tool LDlink 3.6, available at https://ldlink.nci.nih.gov/

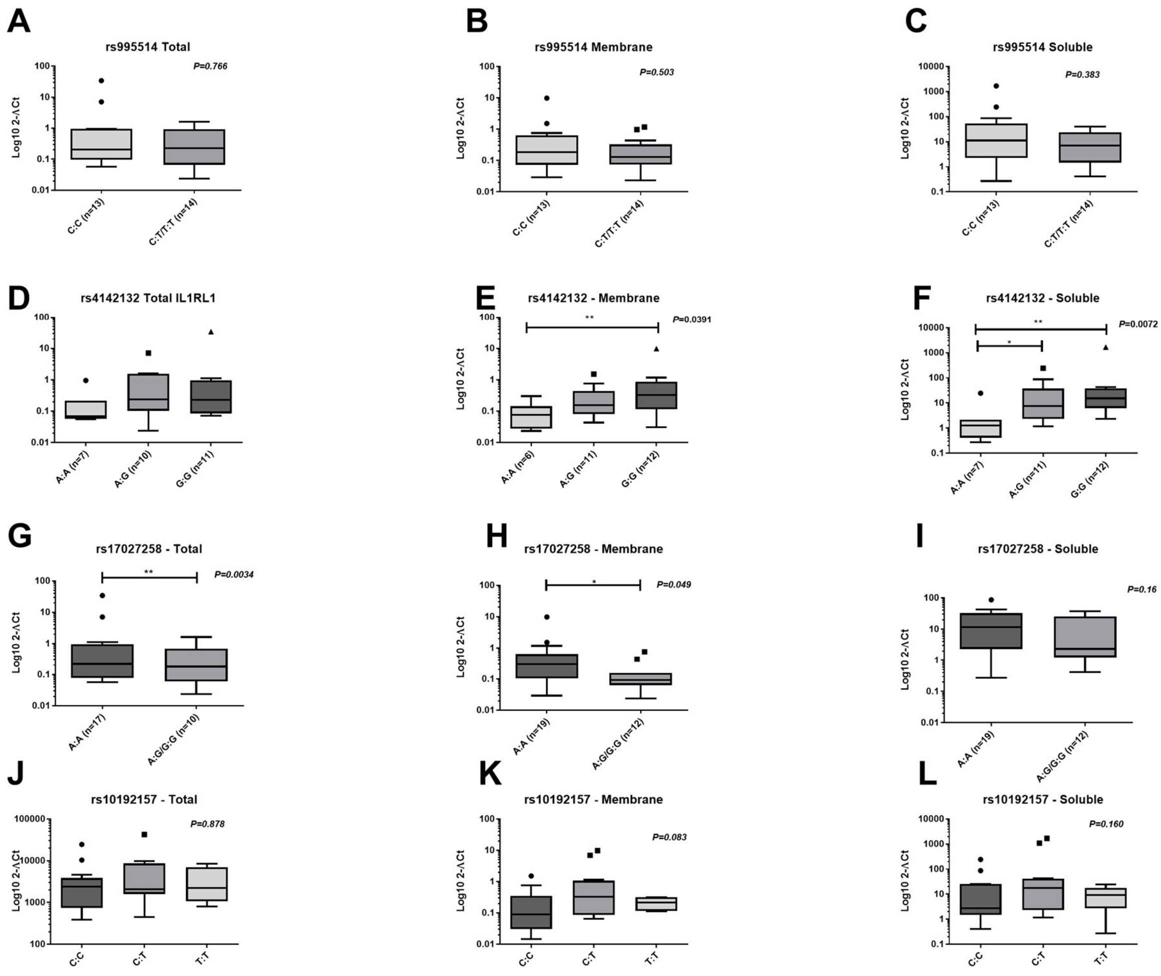

Supplemental Figure 3. *Complete analyses of baseline levels of IL1RL1 mRNA in bronchial epithelial cells* in vitro. *Cells were cultured* in vitro *and IL1RL1 levels were stratified based on the four selected SNPs tagging four loci of association across chromosome 2. Each row represents the mRNA levels (qPCR) of Total – Membrane – Soluble isoforms of IL1RL1 respectively. Panels A-C represent Locus A tagged by rs995514, D-F Locus B tagged by rs4142132, G-I Locus C tagged by rs17027258, J-L Locus D tagged by rs10192157. Statistics run were either Mann-Whitney (Panels A-c, G-I) or Kruskall Wallis (Panels D-F, J-L) as appropriate for two or three group comparisons. *P<0.05, **P<0.01. Data is represented by Tukey box and whisker plots where the box covers data from the 25th to the 75th percentiles with the centre line denoting the median of the data. Whisker plots identify the interquartile range as determined by the Tukey method, with resulting outlier data displayed as distinct points outside the whiskers.*

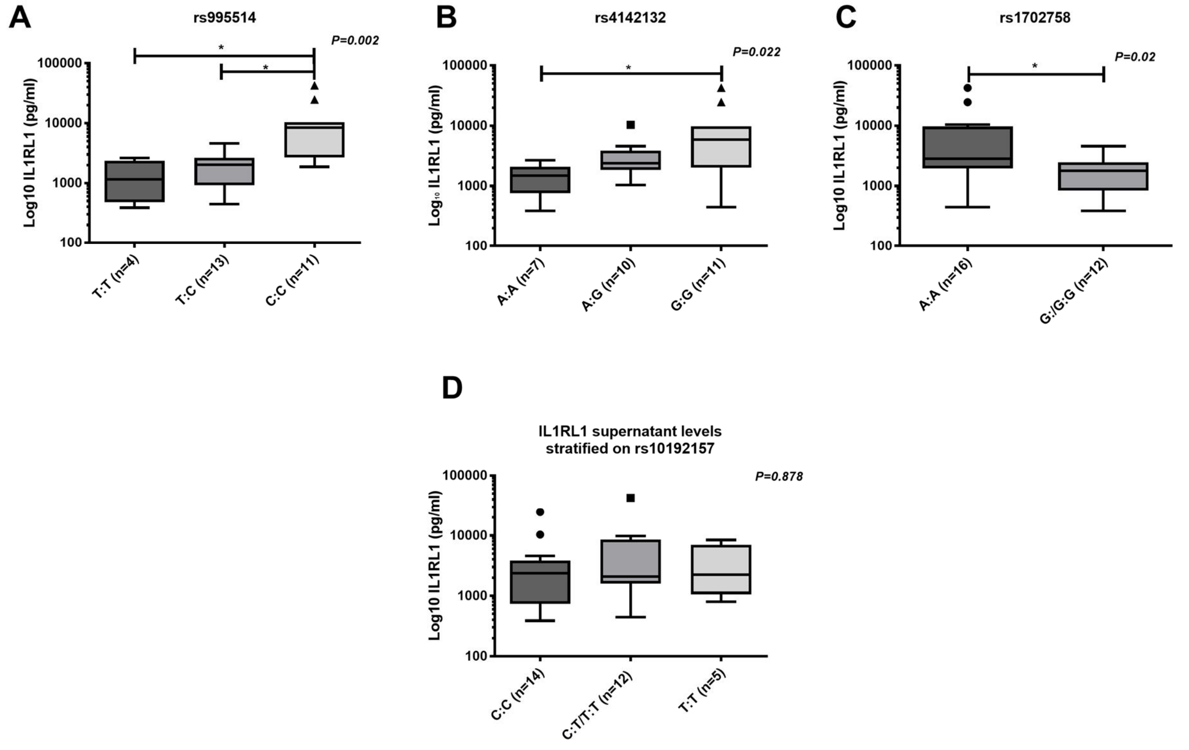

Supplemental Figure 4. Complete analyses of baseline levels of soluble IL1RL1 protein in bronchial epithelial cell supernatants. *Cells were cultured in vitro and protein levels of IL1RL1 (Luminex) were stratified based on the four selected SNPs tagging four loci of association across chromosome 2. Locus A tagged by rs995514 (A), Locus B tagged by rs4142132 (B), Locus C tagged by rs17027258 (C), Locus D tagged by rs10192157 (D). Statistics run were either Mann-Whitney (Panels A, B & D) or Kruskall Wallis (Panel D) as required. *P<0.05, **P<0.01. Data is represented by Tukey box and whisker plots where the box covers data from the 25th to the 75th percentiles with the centre line denoting the median of the data. Whisker plots identify the interquartile range as determined by the Tukey method, with resulting outlier data displayed as distinct points outside the whiskers*

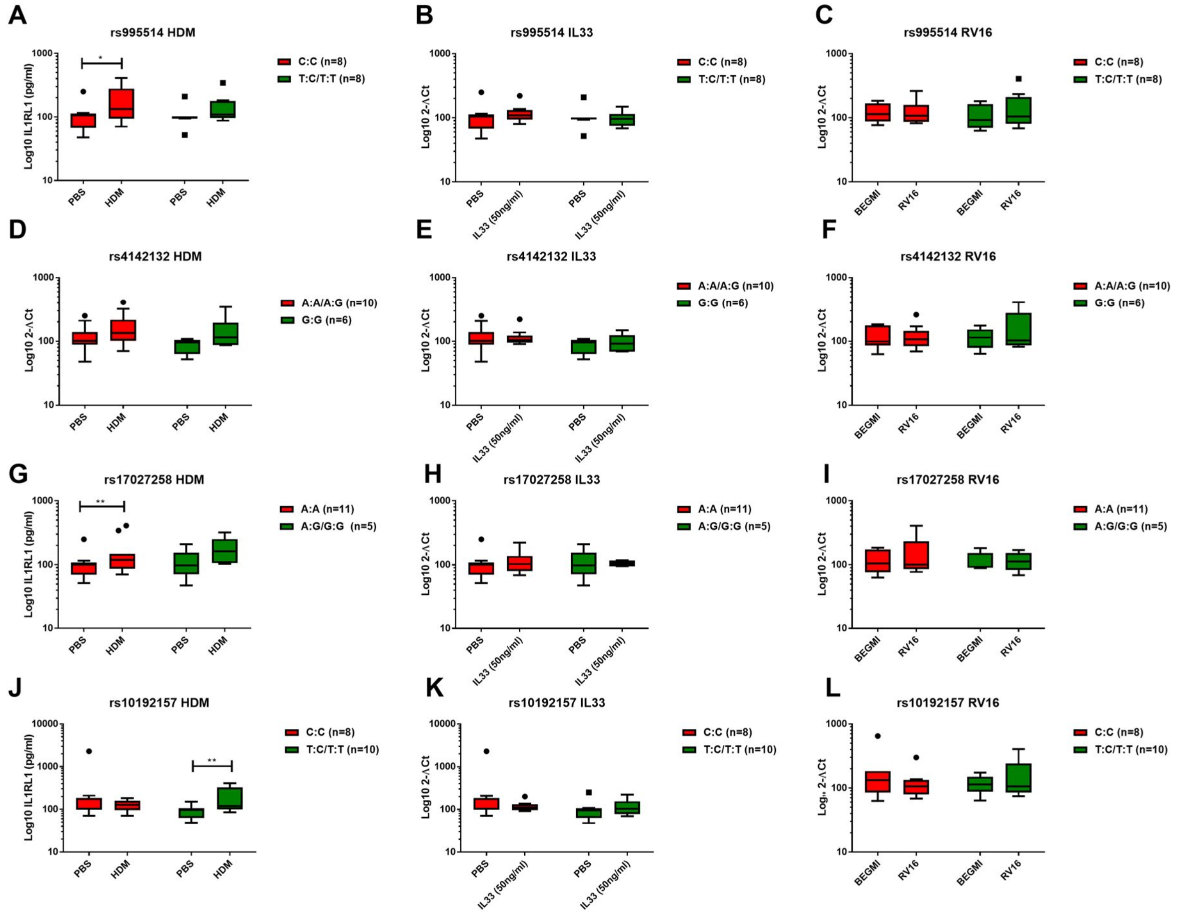

Supplemental Figure 5. Complete analyses of levels of soluble IL1RL1 protein in bronchial epithelial cell supernatants isolated from asthma patients, cultured *in vitro* under different asthma relevant micro-environments and stratified based on the four selected SNPs tagging four loci of association across chromosome 2. Column 1 presents stratification following HDM stimulation, Column 2 following IL33 stimulation (50ng/ml) and Column 3 following RV16 infection (MOI=1). Panels A-C represent Locus A tagged by rs995514, Panels D-F represent Locus B tagged by rs4142132, Panels G-I represent Locus C tagged by rs17027258 and Panels J-L represent Locus D tagged by rs10192157.Statistics were run using Kruskall Wallis tests. *P<0.05, **P<0.01. Data is represented by Tukey box and whisker plots where the box covers data from the 25th to the 75th percentiles with the centre line denoting the median of the data. Whisker plots identify the interquartile range as determined by the Tukey method, with resulting outlier data displayed as distinct points outside the whiskers

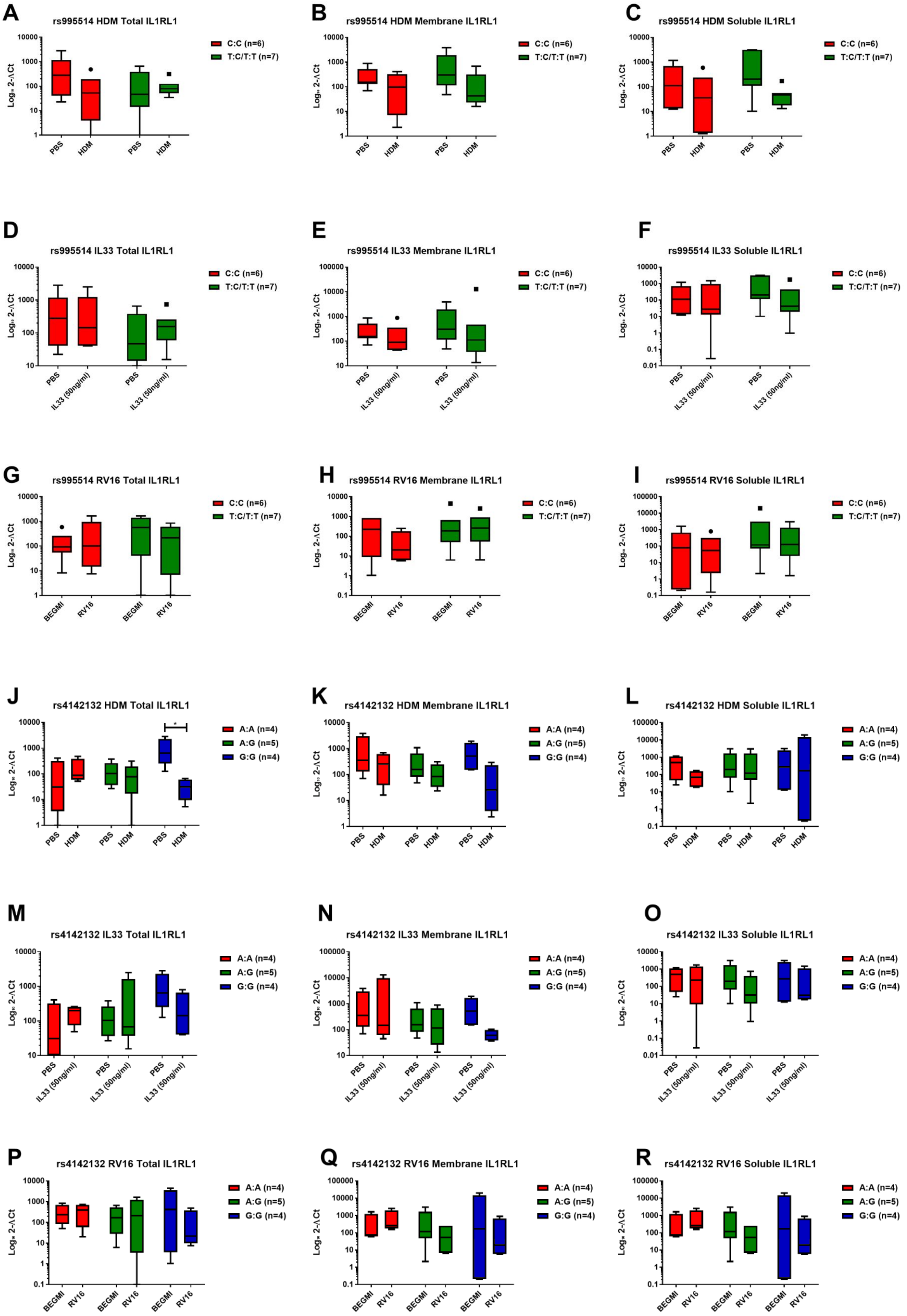
A
rs995514 HDM Total IL1RL1
C:C (n=6)
T:C/T:T (n=7)
B
rs995514 HDM Membrane IL1RL1
C:C (n=6)
T:C/T:T (n=7)
C
rs995514 HDM Soluble IL1RL1
C:C (n=6)
T:C/T:T (n=7)
D
rs995514 IL33 Total IL1RL1
C:C (n=6)
T:C/T:T (n=7)
E
rs995514 IL33 Membrane IL1RL1
C:C (n=6)
T:C/T:T (n=7)
F
rs995514 IL33 Soluble IL1RL1
C:C (n=6)
T:C/T:T (n=7)
G
rs995514 RV16 Total IL1RL1
C:C (n=6)
T:C/T:T (n=7)
H
rs995514 RV16 Membrane IL1RL1
C:C (n=6)
T:C/T:T (n=7)
I
rs995514 RV16 Soluble IL1RL1
C:C (n=6)
T:C/T:T (n=7)
J
rs4142132 HDM Total IL1RL1
A:A (n=4)
A:G (n=5)
G:G (n=4)
K
rs4142132 HDM Membrane IL1RL1
A:A (n=4)
A:G (n=5)
G:G (n=4)
L
rs4142132 HDM Soluble IL1RL1
A:A (n=4)
A:G (n=5)
G:G (n=4)
M
rs4142132 IL33 Total IL1RL1
A:A (n=4)
A:G (n=5)
G:G (n=4)
N
rs4142132 IL33 Membrane IL1RL1
A:A (n=4)
A:G (n=5)
G:G (n=4)
O
rs4142132 IL33 Soluble IL1RL1
A:A (n=4)
A:G (n=5)
G:G (n=4)
P
rs4142132 RV16 Total IL1RL1
A:A (n=4)
A:G (n=5)
G:G (n=4)
Q
rs4142132 RV16 Membrane IL1RL1
A:A (n=4)
A:G (n=5)
G:G (n=4)
R
rs4142132 RV16 Soluble IL1RL1
A:A (n=4)
A:G (n=5)
G:G (n=4)
Log₁₀ 2-ΔCt
PBS
HDM
IL33 (50ng/ml)
BEGMI
RV16

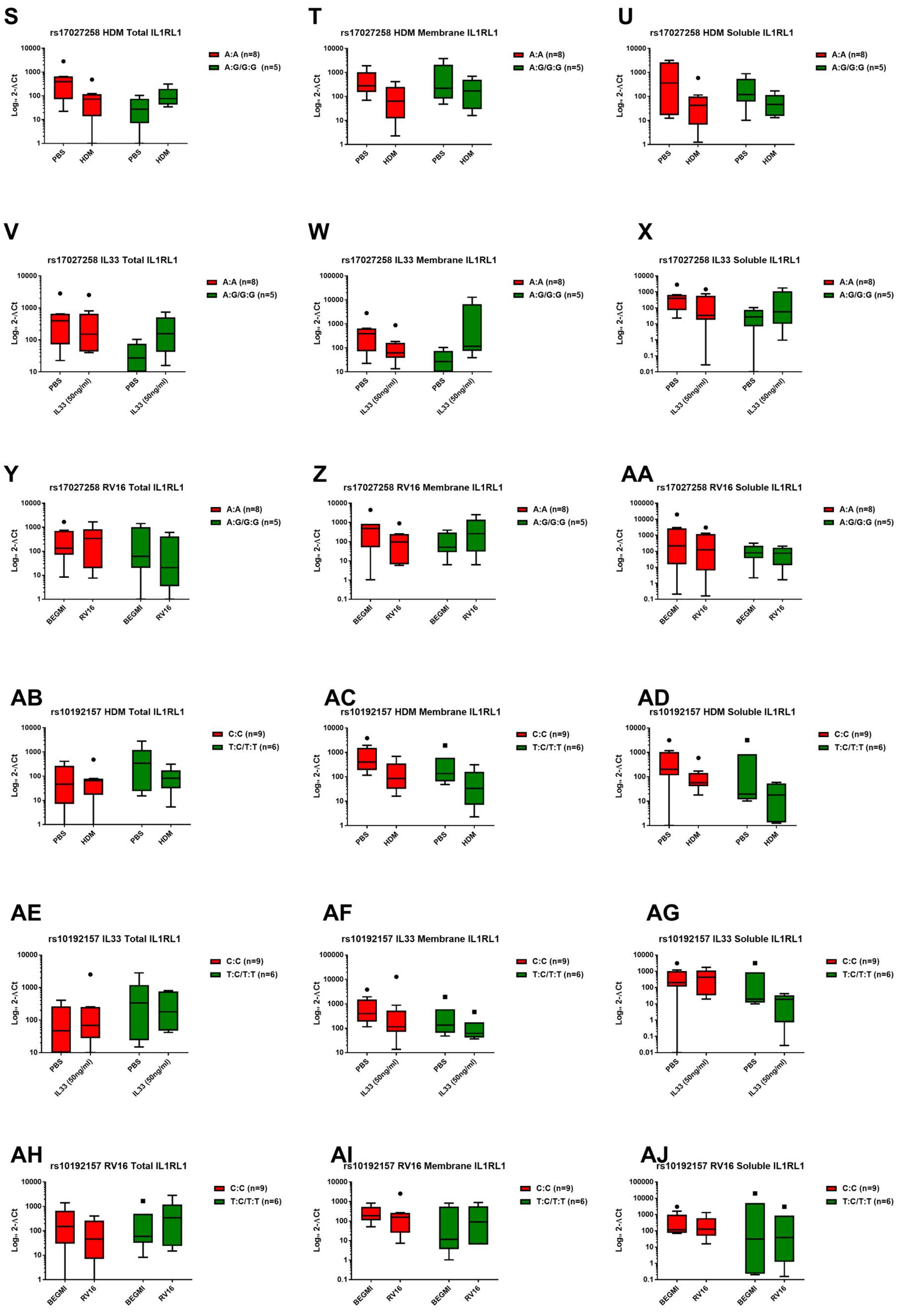
S
rs17027258 HDM Total IL1RL1
A:A (n=8)
A:G/G:G (n=5)
T
rs17027258 HDM Membrane IL1RL1
A:A (n=8)
A:G/G:G (n=5)
U
rs17027258 HDM Soluble IL1RL1
A:A (n=8)
A:G/G:G (n=5)
V
rs17027258 IL33 Total IL1RL1
A:A (n=8)
A:G/G:G (n=5)
W
rs17027258 IL33 Membrane IL1RL1
A:A (n=8)
A:G/G:G (n=5)
X
rs17027258 IL33 Soluble IL1RL1
A:A (n=8)
A:G/G:G (n=5)
Y
rs17027258 RV16 Total IL1RL1
A:A (n=8)
A:G/G:G (n=5)
Z
rs17027258 RV16 Membrane IL1RL1
A:A (n=8)
A:G/G:G (n=5)
AA
rs17027258 RV16 Soluble IL1RL1
A:A (n=8)
A:G/G:G (n=5)
AB
rs10192157 HDM Total IL1RL1
C:C (n=9)
T:C/T:T (n=6)
AC
rs10192157 HDM Membrane IL1RL1
C:C (n=9)
T:C/T:T (n=6)
AD
rs10192157 HDM Soluble IL1RL1
C:C (n=9)
T:C/T:T (n=6)
AE
rs10192157 IL33 Total IL1RL1
C:C (n=9)
T:C/T:T (n=6)
AF
rs10192157 IL33 Membrane IL1RL1
C:C (n=9)
T:C/T:T (n=6)
AG
rs10192157 IL33 Soluble IL1RL1
C:C (n=9)
T:C/T:T (n=6)
AH
rs10192157 RV16 Total IL1RL1
C:C (n=9)
T:C/T:T (n=6)
AI
rs10192157 RV16 Membrane IL1RL1
C:C (n=9)
T:C/T:T (n=6)
AJ
rs10192157 RV16 Soluble IL1RL1
C:C (n=9)
T:C/T:T (n=6)
Log₁₀ 2-ΔCt
PBS
HDM
IL33 (50ng/ml)
BEGMI
RV16

Supplemental Figure 6. Complete analyses of levels of *IL1RL1* mRNA in bronchial epithelial cells isolated from asthma patients and cultured in vitro under different asthma relevant micro-environments then stratified based on the four selected SNPs tagging four loci of association across chromosome 2. Each row represents the mRNA levels of Total – Membrane – Soluble isoforms of IL1RL1, in that order. Panels A-I represent Locus A tagged by rs995514, Panels J-R represent Locus B tagged by rs4142132, Panels S-AA represent Locus C tagged by rs17027258 and Panels AB-AJ represent Locus D tagged by rs10192157. Statistics were run using Kruskall Wallis tests. *$P<0.05$, **$P<0.01$. Data is represented by Tukey box and whisker plots where the box covers data from the 25th to the 75th percentiles with the centre line denoting the median of the data. Whisker plots identify the interquartile range as determined by the Tukey method, with resulting outlier data displayed as distinct points outside the whiskers

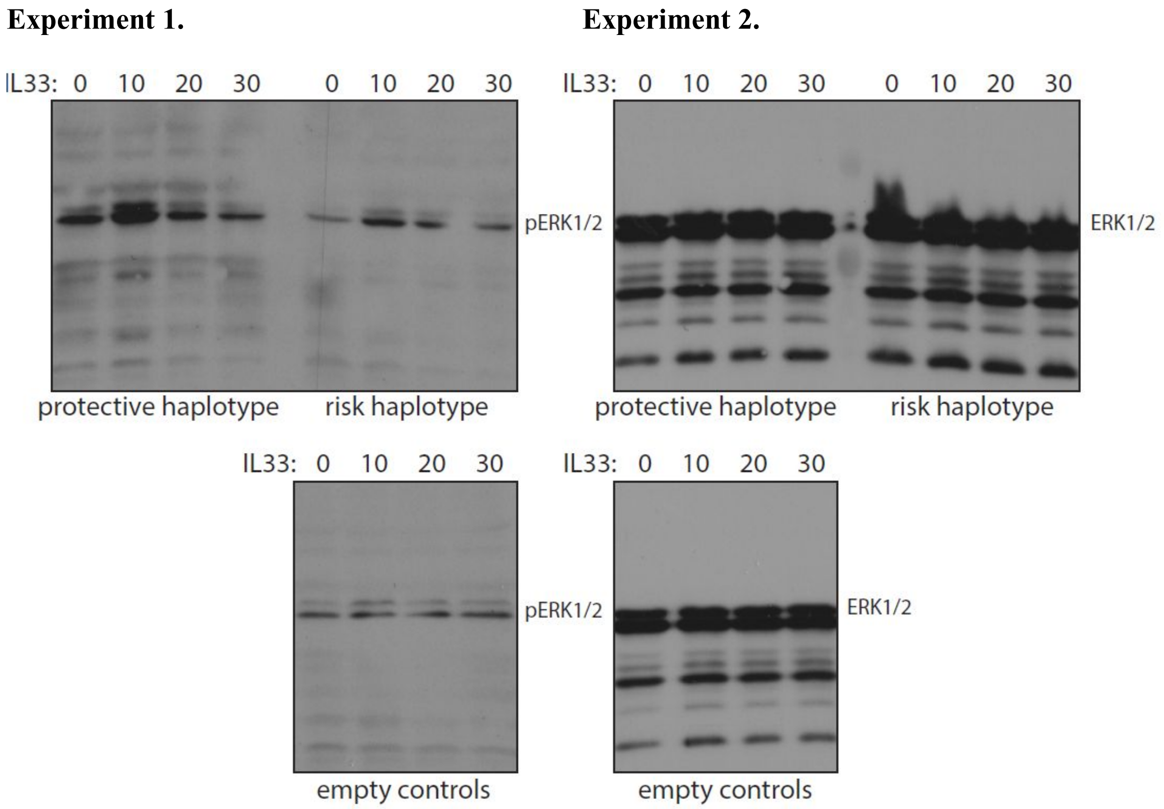

Supplemental Figure 7. Transiently transfected HEK293T cells with *IL1RL1* protective or risk haplotype. Cells were stimulated with recombinant IL33 (30 ng/ml) for 0, 10, 20 or 30 minutes as indicated followed by lysis and Western blot analysis for total and phosphorylated ERK1/2. HEK293T cells transfected with either *IL1RL1* haplotype display an IL33 induced phosphorylation of ERK that is largely absent in empty control cells.

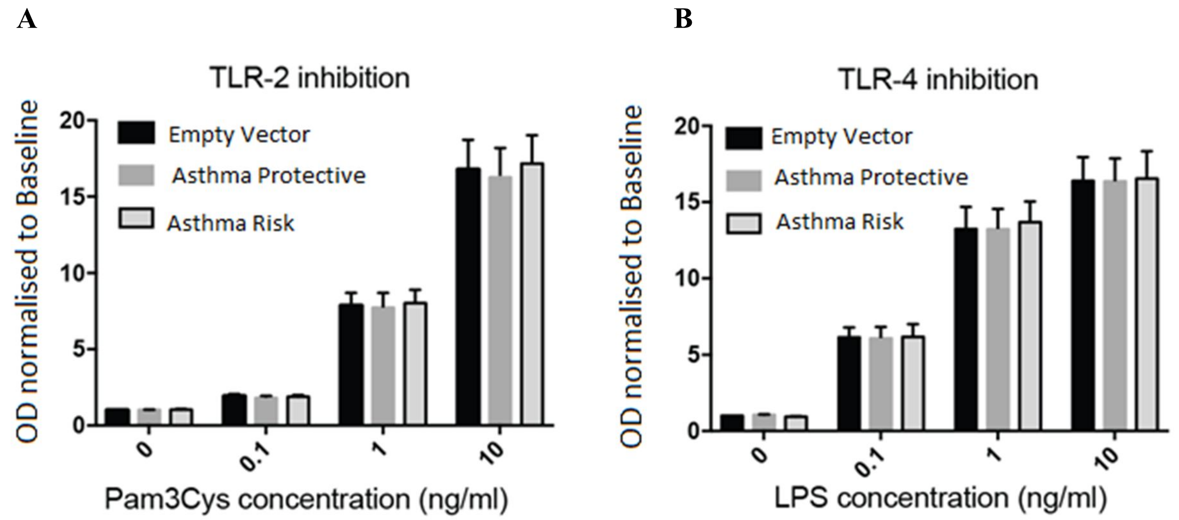

Supplemental Figure 8. **Normalized SEAP (NF-κB) activity assay in TLR2 and TLR4 reporter transfected HEK cells.** TLR2 and TLR4 expressing HEK cells transiently transfected with pCDNA3.1 empty vector (negative control) or encoding the asthma risk or non-risk IL1RL1 Ala433Thr/Glu501Arg/Thr549Ile/Leu551Ser haplotypes were stimulated with a dose range of PAM3Cys (Panel A) or LPS (Panel B), respectively. Mean values are presented for cells transiently transfected with the empty vector, Bars represent mean+/- Standard Error of the mean.

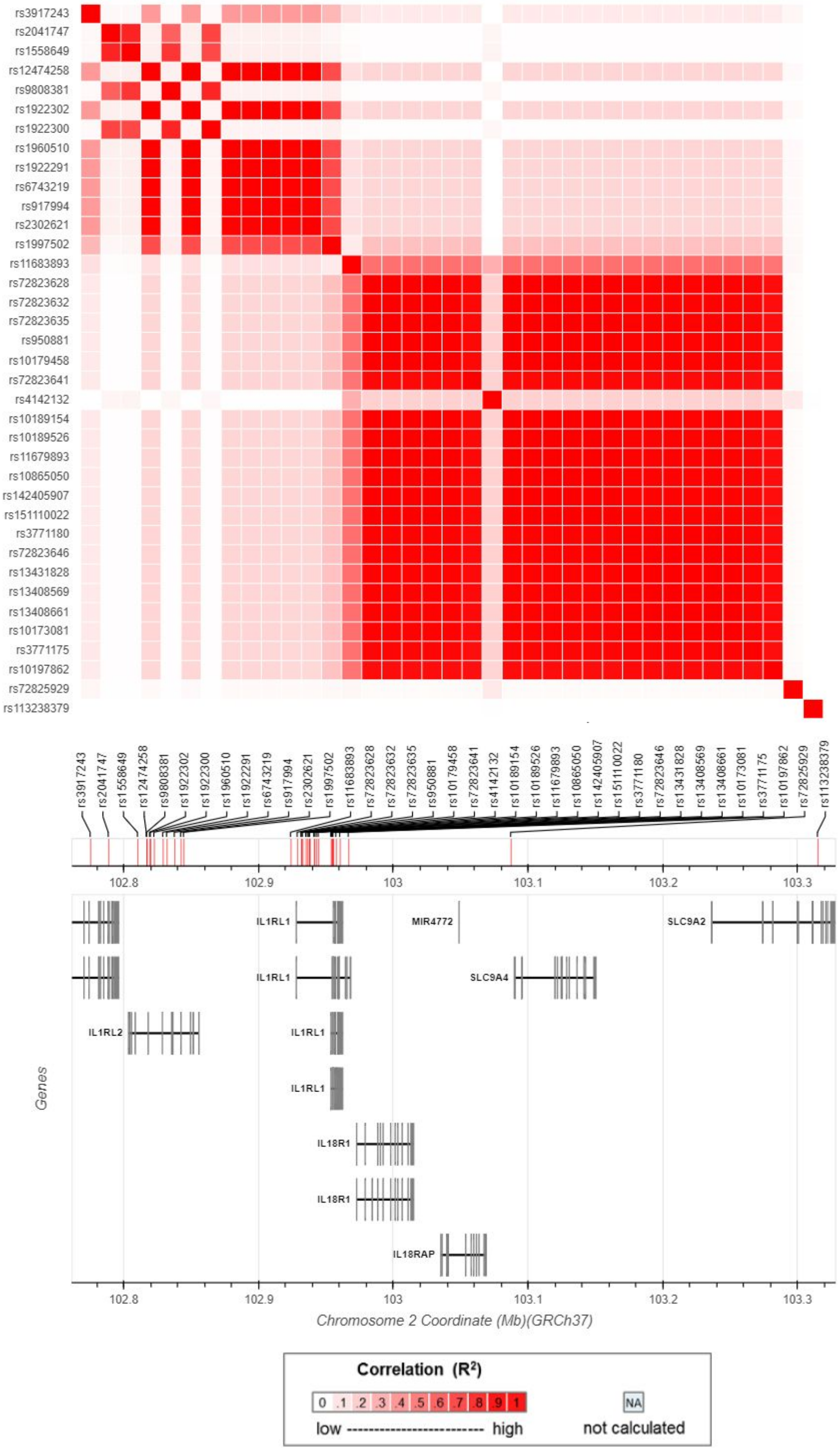

Supplemental Figure 9: Linkage disequilibrium map of chromosome 2 illustrating LD patterns of the three detected Signals (A-C) associated with one or more trait in the association analyses (Stage 1). Figure identifies the level of LD between signals identified based on r^2. Image generated using the EUR population of the Phase I cohort of the 1000 genomes study via the online software tool LDlink 3.0, available at: https://analysistools.nci.nih.gov/LDlink/?tab=home

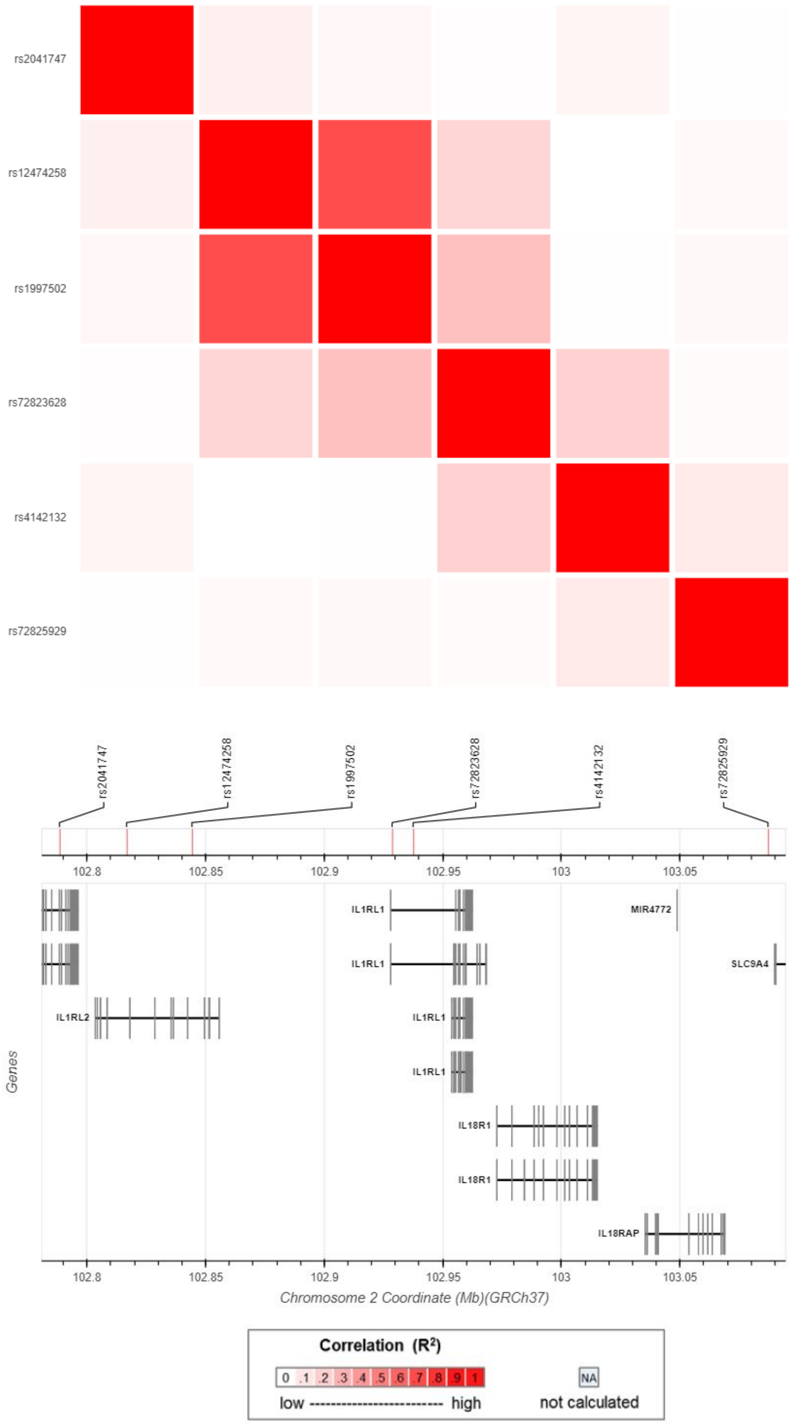

Supplemental Figure 10: Linkage disequilibrium map of chromosome 2 illustrating location of 6 variants prioritised through Stage 2 SNP selection. Figure identifies the level of LD between signals identified based on r^2. Image generated using the EUR population of the Phase I cohort of the 1000 genomes study via the online software tool LDlink 3.0, available at: https://analysistools.nci.nih.gov/LDlink/?tab=home

Supplemental Tables

Please see attached Excel File for Supplemental Tables 1-7.

Supplemental Table 1. Resequencing of the chromosome 2 locus. Region based resequencing of 200 UK severe asthma cases and 200 UK non-asthmatic, non-atopic controls identifies 4108 variants within the region of which fifteen represent *IL1RL1* coding region variation, rs13431828, rs201837678, rs202037442, rs1041973, rs111970215, rs138892317, rs4988956, rs111942110, rs4988957, rs10192036, rs10204137, rs138992262, rs4988958, rs10192157, rs10206753. Case-control association analyses for variant association with diseases state. Using FDR<0.05 we identify 71 variants that are associated with disease status (SNVer). OR, Odds ratio

Supplemental Table 2. Variants achieving FDR<0.05 from the resequencing of the chromosome 2 locus. Eight *IL1RL1* coding variants achieve FDR<0.05 with disease status (SNVer), based off region based resequencing of 200 UK severe asthma cases and 200 UK non-asthmatic, non-atopic controls. Variants were identified based on case-control association analyses for variant association with diseases state. OR, Odds ratio

Supplemental Table 3. Exon resequencing of *IL1RL1*. Data identifies 56 variants spanning the *IL1RL1* introns, exons and promoter region. MAF, Minor allele frequency.

Supplemental Table 4. Association results for all variants meeting initial criteria (FDR<0.05) for association with asthma diagnosis in Lifelines. A1: Coding Allele, BP: Basepair position, FRQ: Coding allele frequency in cohort, SE: standard error.

Supplemental Table 5. Association results for all variants meeting initial criteria (FDR<0.05) for association with blood eosinophil levels (General Population) in Lifelines. A1: Coding Allele, BP: Basepair position, FRQ: Coding allele frequency in cohort, SE: standard error.

Supplemental Table 6. Association results for all variants meeting initial criteria (FDR<0.05) for association with atopy as defined by a positive response to a skin prick test in a combined meta-analysis of the GASP and DAG cohorts. MAF, Minor allele frequency; SE, standard error.

Supplemental Table 7. Association results for all variants meeting initial criteria (FDR<0.05) for association with lung function as defined by FEV_1 in a combined meta-analysis of the GASP and DAG cohorts. Allele 1: Coding Allele, Effect: Beta

Supplemental Table 8. Association results of chromosome 2 variation previously reported to be associated with asthma. List of chromosome 2 polymorphisms published in the literature as showing association to asthma traits. Among the asthma literature SNPs, statistically significant association of multiple SNPs in our datasets are noted for both asthma and blood eosinophil counts in Lifelines (FDR<0.05).

*SNP also associated with blood eosinophil counts in literature (27).

SNP	POSITION (BP)	GENE	VARIANT ALLELES	ANCESTRAL ALLELE	ASSOCIATION	FDR	EFFECT	SE	PMID
rs1558641	102765865	5' of IL1RL1	G/A	G	Eos GP Lifelines	0.038	0.03	0.01	26493291
rs202011557	102913643	5' of IL1RL1	-/AAAC/AAACAAAC/AAC	-	-	-	-	-	27182965
rs11685480	102927086	5' of IL1RL1	G/A	-	-	-	-	-	21629437<br>27699235<br>21281963<br>22574108<br>24568840
rs6543116	102927726	5' of IL1RL1	A/G	A	-	-	-	-	19852851<br>21629437<br>24568840<br>19254249<br>16118232
rs12479210	102949161	IL1RL1	C/A/T	T	Eos GP Lifelines	0.012	0.03	0.008	30552067<br>30578877
rs13431828	102954653	IL1RL1	C/T	C	Asthma Lifelines	0.04909	1.35	0.08	24568840<br>22357570<br>20816195<br>20860503<br>19910030<br>22694930<br>21629437<br>25932636<br>23209423
rs1041973	102955468	IL1RL1	C/A	A	-	-	-	-	19910030<br>19852851<br>28266165

									27699235<br>21281963<br>22574108<br>28273074<br>25091434<br>23999434<br>20816195<br>20860503
rs10173081	102957348	IL1RL1	C/T	T	Asthma Lifelines	0.04909	1.35	0.08	23028483<br>21804549<br>27699235<br>26102239
rs1420101*	102957716	IL1RL1	C/T	T	Eos GP Lifelines	0.012	0.03	0.009	18774397<br>21738479<br>19910030<br>23028483<br>21150878<br>20833654<br>19852851<br>26069107<br>21629437<br>27699235<br>21281963<br>22574108<br>21966603<br>20592918<br>25091434<br>24568840<br>20816195<br>20860503<br>19198610
rs1921622	102966067	IL1RL1	G/A	A	-	-	-	-	18774397<br>22357570<br>19852851<br>21629437<br>27699235

									25091434
rs10208293	102966310	IL1RL1	G/A	A	Asthma Lifelines<br>Eos GP Lifelines	0.04909<br>0.012	1.22<br>0.03	0.06<br>0.01	27130862<br>21629437<br>24568840
rs10197862	102966549	IL1RL1	A/G	A	Asthma Lifelines	0.04909	1.34	0.08	22357570<br>23028483<br>21150878<br>27058054
rs1861245	102966906	IL1RL1	C/T	C	-	-	-	-	19852851<br>21629437
rs13424006	102967236	IL1RL1	T/C	C	Asthma Lifelines<br>Eos GP Lifelines	0.04909<br>0.028	1.18<br>0.03	0.05<br>0.008	24568840
rs4988958	102968285	IL1RL1	T/C	T	Asthma Lifelines<br>Eos GP Lifelines	0.04909<br>0.028	1.18<br>0.03	0.05<br>0.008	22357570
rs10192157	102968356	IL1RL1	C/T	C	Asthma Lifelines<br>Eos GP Lifelines	-0.04909<br>0.028	1.18<br>0.03	0.05<br>0.008	20816195<br>20860503<br>21150878<br>25409453<br>27058054
rs9807989	102971200	3` of IL1RL1	T/C	T	Eos GP Lifelines	0.029	0.03	0.008	23755072<br>22561531
rs3771166	102986222	IL18R1	G/A/T	A	Eos GP Lifelines	0.033	0.02	0.008	19852851<br>19910030<br>23886662<br>20860503<br>23028483<br>23755072<br>21543792<br>27130862<br>27658857<br>21629437<br>21966603
rs2310300	103049074	IL18RAP	A/G	A	Asthma Lifelines<br>Eos GP Lifelines	0.04909<br>0.036	1.16<br>0.02	0.05<br>0.008	20860503<br>22846752

Supplemental Table 9. Detailed ENCODE analyses of four priority tagging SNPs identified several putative regulatory mechanisms.

Detailed ENCODE analyses of the four selected SNPs tagging our 4 regions of association, identified several putative regulatory mechanisms. LD, linkage disequilibrium.

*LD is based on r2>0.8, †Evidence of IL1RL1 eQTL in lung tissue.

TAGGING SNP	SNPS IN LD*	ENHANCER HISTONE MARKS IN LUNG	DNASE IN LUNG	PROTEINS BOUND	MOTIFS CHANGED
**rs12474258**	6	Y	N	CTCF, RAD21, MAFK, HAE2F1	BRACHYURY, GATA, MYF, ZFP105, AP-1, HLX1, IRF, NKX6-1, POU3F1, STAT, SP100, TCF12
**rs4142132**	62	Y	Y	CTCF, IRF4, RAS21, SMC3, GATA2, SETDB1, MAFK, STAT3	IRF, AFP1, AHR::ARNT, AHR::ARNT::HIF1, AIRE, AP-1, AP-2, ARID3A, ARID5A, ASCL2, ATF3, BARHL1, BBX, BCL, BDP1, CART1, CDP, CDX2, CEBPB, CIZ, CTCF, DBX1, DBX2, DMRT2, DMRT3, DMRT7, EN-1, ESX1, ETS, EVI-1, EWSR1-FLI1, FAC1, FOX, FOXA, FOXD3, FOXF1, FOXI1, FOXJ2, FOXL1, FOXO, FOXP1, FOXQ1, FXR, GFI1, GLI, GM397, GR, HAND1, HAND1, HDAC2, HDX, HIF1, HMG-IY, HNF1, HNF4, HNF6, HOXA10, HOXA3, HOXA4, HOXA5, HOXA9, HOXB13, HOXB9, HOXC6, HSF, IK-2, IRC900814, IRF, IRX, ISL2, ISX, LF-A1, LHX4, MAF, MEF2, MRG, MSX-1, MYF, NANOG, NCX, NF-I, NF-KAPPAB, NKX2, NKX3, NKX6-1, NRF-2, NRSF, P300, PAX-4, PAX-5, PAX-6, PAX7, PBX-1, PBX3, PDX1, PHOX2A, PITX2, PLZF, POU1F1, POU2F2, POU3F2, POU3F3, POU3F4, POU4F3, POU5F1, POU6F1, PRDM1, PRRX1, PRRX2, PU.1, RAD21, RAR, RFX5, RREB-1, RXRA, SMAD, SMAD3, SMC3, SOX, STAT, TAL1, TATA, TCF12, TCF4, TGIF1, TLX1::NFIC, VAX2, VDR, YY1, ZBTB12, ZFP105, ZFP161, ZID
**rs72825929**	0	N	N	NONE	SOX
**rs10192157**	49	N	N	GATA2, EGR1,POL2, CTCF,SMC3,CEBPB	CTCFL,CTCF,NRSF,PTF1-BETA,STAT,ZNF263, GR,SMAD,YY1, MEF2,TATA,MEF2,RHOX11, FOXA,HDX

		,POU2F2, BCL,PRDM1,PU.1,PAX-5,RXRA,NFAT,PRDM1,PU.1,STAT,BCL,PRDM1,PU.1,PAX5, CDX,NKX2,NKX3,SPDEF, ZBTB3, AP-1,FAC1,P300,AP1,FAC1,P300,AP3,GATA,POU3F1,PRDM1,PAX-5,ROAZ,POU2F2,ROAZ,OBOX6, DUXL,ATF3,ATF6,NKX2,FOX,FOXC1,FOXD3,FOXF1,FOXF2,FOXI1,FOXJ1,FOXJ2,FOXK1,FOXL1,FOXO,FOXQ1,HDAC2,SOX,ZFP105,CDX2,DMRT4,DBX1,HNF1,HOXD10,NCX,NKX6-1,PAX4,POU2F2,SOX, ,TEF,CDC5,GR, HOXA5,CDP,ZFP691,FAC1, CDP, ,GR,HMG-IY,RREB-1,SOX,ZFP105, ERALPHA-A,GFI1,LRH1,BACH2,CDX,HOXA5,IRF,SIX5, ARID3A,BARHL1,CART1,DBX1,DBX2,EN-1,FOXA,GR,HOXA7,HOXB4,HOXB9,ISL2,MSX-1,NCX,NKX6-1,NKX6-2,PAX-4,PAX7,POU1F1,POU3F4,POU4F3,SOX, CEBPB,ERALPHA-A,PLZF,RAR,RXRA,VDR, AHR,HEY1, GR, ESR2, EBF,SZF1-1,ZEB1, CART1, IRF,POU2F2, IRF,POU2F2, HDAC2,IK-2,NF-AT1,POU2F2,STAT, FOXP3,IRF,STAT, ARID5B,MRG,PLZF,RXR::LXR, HNF1,HNF4,IRF,PAX-4,RREB-1, PU.1,SIN3AK-20, RREB-1,UF1H3BETA, CDP,GR,HNF1, HNF1,IK-2, BBX,POU5F1,SIX5

Supplemental Table 10. Demographics of the GASP, DAG and Lifelines cohorts used independently and together (GASP & DAG) for genetic association analyses. Percentage values are presented as percent of valid subjects.

Characteristics	GASP (N=2536)	N	DAG (N=909)	N	Lifelines Asthma (N=1066)	N	Lifelines Control (N=6863)	N
Age (yrs), mean (SD)	47.83 (15.51)	2285	34.78 (15.80)	909	46.2 (10.9)	1066	49.2 (11.9)	6863
Gender, Male (%)	36.0	2534	46.9	909	40.2	1066	46.8	6863
Height (m), mean (SD)	1.66 (0.10)	2095	1.68 (0.16)	905	1.80 (0.10)	1066	1.75 (0.09)	6863
FEV_1 (L), mean (SD)	2.26 (0.85)	2089	2.81 (0.94)	899	3.1 (0.8)	1066	3.4 (0.8)	6863
FEV_1/FVC, mean (SD)	0.69 (0.14)	1931	0.79 (0.10)	262	0.73 (0.09)	1066	0.77 (0.7)	6863
Blood Eosinophils (10-9/L), median (range)	0.31 (0.00-5.42)	1018	0.23 (0.00-1.90)	769	0.20 (0.13-0.30)	1066	0.15 (1.10-0.22)	6863
Total IgE (kU/L), median (range)	406.79 (1.00 – 5000.00)	1379	378.41 (0.00 – 12400.00)	772	ND	ND	ND	ND
Atopy* (%)	1072 (68.5)	1559	578 (85.4)	677	ND	ND	ND	ND
Age of asthma onset (yrs), mean (SD)	23.20 (17.95)	1178	10.07 (10.58)	689	ND	ND	ND	ND
Childhood onset asthma†	578 (46.2)	1284	520 (75.5)	689	ND	ND	ND	ND

N = number of subjects data field available for, SD: Standard of Deviation, ND = Not Determined/Defined

*Atopy was based on at least one positive response to intracutaneous or skin prick tests (SPT) †Childhood onset asthma defined as a diagnosis occurring before the age of 16.

Supplemental Table 11. Demographics for the asthma patients taken from DAG (95 cases) used in the next-generation sequencing of the chromosome 2 locus.

Characteristics	DAG (n=95)	N
Age (yrs), mean (SD)	33.89 (14.5)	95
Gender, Male (%)	36.8%	95
Height (m), mean (SD)	1.74 (0.09)	95
Smoking (%)		
Current	10.6%	94
Ex	33.0%	
Never	56.4%	
Smoking (pky)	8.67 (6.8)	40
Steroid use		
ICS only	80%	95
ICS+OCS	2.1%	
No CS	17.9%	
Reversibility (%pred) qual		
>9	43.6%	94
<9	56.4%	
FEV_1 prebd (L), mean (SD)	3.29 (0.74)	94
%pred	90.3%	
FEV_1/FVC, mean (SD)	0.77 (0.11)	94
Blood Eosinophils (10-9/L), median (range)	0.16 (0.001-0.64)	95
Total IgE (kU/L), median (range)	504.63 (3.0-8650.0)	95
Atopy* (%)	79 (84.9%)	93
Age of asthma onset (yrs), mean (SD)	-	-
Childhood onset asthma†	-	-

N = number of subjects data field available for, SD: Standard of Deviation, ND = Not Determined/Defined. *Atopy was based on at least one positive response to intracutaneous or skin prick tests (SPT) †Childhood onset asthma defined as a diagnosis occurring before the age of 16.

Supplemental Table 12. Demographics for the sub-cohorts taken from GASP (200 cases) and GEDLING (5) (200 non-asthmatic, non-atopic controls) used in the next-generation sequencing of the chromosome 2 locus.

Characteristics	GASP Cohort (cases)	Gedling Cohort (controls)
Age (yrs), mean (SD)	48 (14.88)	57 (12.64)
Gender, Male (%)	30.6	27.0
Height (m), mean (SD)	1.64 (0.08)	1.66 (0.06)
FEV_1 (L), mean (SD	2.17 (0.84)	2.77 (0.79)
Smoking pack/years	11.82 (20.25)	8.40 (18.61)
Never Smokers (%)	52.0	53.5

Supplemental Table 13. Demographics of the Manchester Asthma and Allergy Study at age 8 years

Characteristics	MAAS (N=919)	N
Age (yrs), mean (SD)	7.98 (0.16)	919
Gender, Male (%)	53.8	919
Height (cm) , mean (SD)	128.2 (5.61)	919
FEV_1 (L), mean (SD)	1.59 (0.25)	695
FEV_1/FVC, mean (SD)	0.87 (0.06)	695
Atopy, number (%)	32.9	817
Asthma, number (%)	23.9	872

Supplemental Table 14. Expression quantitative trait loci (eQTL) analysis for tagging SNPs or proxies in a dataset in bronchial biopsy (n=77) and bronchial brushes (n=72) from healthy individuals.

			Bronchial biopsy			Bronchial brushing		
**Signal/SNP**	**(C/NC*)**	**MAF**	**Beta**	**SE**	**Pval**	**Beta**	**SE**	**Pval**
A rs10167431†	G/A	0.44	-0.01	0.17	0.93	-0.01	0.06	0.87
B rs10178436††	G/A	0.45	0.01	0.19	0.97	-0.07	0.06	0.29
C rs2241116†††	C/A	0.17	-0.11	0.25	0.66	0.04	0.07	0.62
D rs10192157	G/A	0.32	-0.09	0.16	0.55	-0.02	0.05	0.64

*SNPs are reported in relation to the linkage disequilibrium (LD) blocks (r^2>0.8) reported in this study.

TAO: Time to asthma onset

†Variants relate to the variants subsequently selected as tagging SNPs for functional studies.

No SNPs were identified as eQTLs in this dataset. *C=coded allele, NC=Non-coded allele, †rs10167431 was used as a proxy for rs12474258 (r2=0.68), ††rs10178436 was used as a proxy for rs4142132 (r2=0.98), †††rs2241116 was used as a proxy for rs72825929 (r2=0.37), MAF= Minor allele frequency

Supplemental Table 15. Demographics for donors of bronchial epithelial cells contributing to the AHBEC dataset.

Characteristics	AHBEC (N=51)	N
Age (yrs), mean (SD)	50 (13.47)	20
Gender, Male (%)	43.5	23
Height (m), mean (SD)	1.71 (0.10)	13
FEV_1 (L), mean (SD)	2.70 (0.95)	25
FEV_1/FVC , mean (SD)	0.69 (0.11)	19
Atopy*, number (%)	7 (58.3)	12

*Atopy was defined as a positive response to a skin prick test. Data was not available for the full cohort of 51 individuals.

Supplemental References

1. Nick Shrine MAP, Catherine John, Maria Soler Artigas, Neil Bennett Robert Hall, Jon Lewis, Amanda P Henry, Charlotte K Billington, Azaz Ahmad, Richard J Packer, Dominick Shaw, Zara E Pogson, Andrew Fogarty, Tricia M McKeever, Amisha Singapuri Liam G Heaney, Adel H Mansur, Rekha Chaudhuri, Neil C Thomson, John W Holloway, Gabrielle A Lockett, Peter H Howarth, Ratko Djukanovic, Jenny Hankinson, Robert Niven, Angela Simpson, Kian Fan Chung, Peter J Sterk, John D Blakey, Ian M Adcock, Sile Hu, Yike Guo, Maen Obeidat,Don D Sin, Maarten van den Berge, David C Nickle, Yohan Bossé, Martin D Tobin, Ian P Hall, Christopher E Brightling, Louise V Wain, Ian Sayers. A genome-wide association study of moderate-severe asthma in individuals of European ancestry. Lancet Respiratory Medicine. 2018;(In Press).
2. Nieuwenhuis MA, Siedlinski M, van den Berge M, Granell R, Li X, Niens M, et al. Combining genomewide association study and lung eQTL analysis provides evidence for novel genes associated with asthma. Allergy. 2016;71(12):1712-20.
3. Custovic A, Simpson BM, Murray CS, Lowe L, Woodcock A, Asthma NACM, et al. The National Asthma Campaign Manchester Asthma and Allergy Study. Pediatr Allergy Immunol. 2002;13 Suppl 15:32-7.
4. Scholtens S, Smidt N, Swertz MA, Bakker SJ, Dotinga A, Vonk JM, et al. Cohort Profile: LifeLines, a three-generation cohort study and biobank. Int J Epidemiol. 2015;44(4):1172-80.
5. McKeever TM, Lewis SA, Smith C, Collins J, Heatlie H, Frischer M, et al. Siblings, multiple births, and the incidence of allergic disease: a birth cohort study using the West Midlands general practice research database. Thorax. 2001;56(10):758-62.
6. BTS/SIGN. BTS/SIGN Asthma Guideline 2016 2016 [Available from: https://www.brit-thoracic.org.uk/document-library/clinical-information/asthma/btssign-asthma-guideline-2016/.
7. Standardization of Spirometry, 1994 Update. American Thoracic Society. Am J Respir Crit Care Med. 1995;152(3):1107-36.
8. Hao K, Bosse Y, Nickle DC, Pare PD, Postma DS, Laviolette M, et al. Lung eQTLs to help reveal the molecular underpinnings of asthma. PLoS Genet. 2012;8(11):e1003029.
9. Boudewijn IM, Postma DS, Telenga ED, Ten Hacken NH, Timens W, Oudkerk M, et al. Effects of ageing and smoking on pulmonary computed tomography scans using parametric response mapping. Eur Respir J. 2015;46(4):1193-6.
10. Imkamp K, Berg M, Vermeulen CJ, Heijink IH, Guryev V, Kerstjens HAM, et al. Nasal epithelium as a proxy for bronchial epithelium for smoking-induced gene expression and expression Quantitative Trait Loci. J Allergy Clin Immunol. 2018;142(1):314-7 e15.
11. Du Rand IA, Blaikley J, Booton R, Chaudhuri N, Gupta V, Khalid S, et al. British Thoracic Society guideline for diagnostic flexible bronchoscopy in adults: accredited by NICE. Thorax. 2013;68 Suppl 1:i1-i44.
12. Shaw DE, Sousa AR, Fowler SJ, Fleming LJ, Roberts G, Corfield J, et al. Clinical and inflammatory characteristics of the European U-BIOPRED adult severe asthma cohort. Eur Respir J. 2015;46(5):1308-21.
13. Slater M, Torr E, Harrison T, Forrester D, Knox A, Shaw D, et al. The differential effects of azithromycin on the airway epithelium in vitro and in vivo. Physiol Rep. 2016;4(18).
14. Portelli MA, Stewart CE, Hall IP, Brightling CE, Sayers I. Cigarette Smoke and the Induction of Urokinase Plasminogen Activator Receptor In Vivo: Selective Contribution of Isoforms to Bronchial Epithelial Phenotype. Am J Respir Cell Mol Biol. 2015;53(2):174-83.
15. Price AL, Patterson NJ, Plenge RM, Weinblatt ME, Shadick NA, Reich D. Principal components analysis corrects for stratification in genome-wide association studies. Nat Genet. 2006;38(8):904-9.
16. Genomes Project C, Abecasis GR, Auton A, Brooks LD, DePristo MA, Durbin RM, et al. An integrated map of genetic variation from 1,092 human genomes. Nature. 2012;491(7422):56-65.
17. Moffatt MF, Gut IG, Demenais F, Strachan DP, Bouzigon E, Heath S, et al. A large-scale, consortium-based genomewide association study of asthma. N Engl J Med. 2010;363(13):1211-21.

18. Torgerson DG, Ampleford EJ, Chiu GY, Gauderman WJ, Gignoux CR, Graves PE, et al. Meta-analysis of genome-wide association studies of asthma in ethnically diverse North American populations. Nat Genet. 2011;43(9):887-92.
19. Li H, Durbin R. Fast and accurate short read alignment with Burrows-Wheeler transform. Bioinformatics. 2009;25(14):1754-60.
20. DePristo MA, Banks E, Poplin R, Garimella KV, Maguire JR, Hartl C, et al. A framework for variation discovery and genotyping using next-generation DNA sequencing data. Nat Genet. 2011;43(5):491-8.
21. Rivas MA, Beaudoin M, Gardet A, Stevens C, Sharma Y, Zhang CK, et al. Deep resequencing of GWAS loci identifies independent rare variants associated with inflammatory bowel disease. Nat Genet. 2011;43(11):1066-73.
22. Faiz A, van den Berge M, Vermeulen CJ, Ten Hacken NHT, Guryev V, Pouwels SD. AGER expression and alternative splicing in bronchial biopsies of smokers and never smokers. Respir Res. 2019;20(1):70.
23. Kim D, Langmead B, Salzberg SL. HISAT: a fast spliced aligner with low memory requirements. Nat Methods. 2015;12(4):357-60.
24. Li H, Handsaker B, Wysoker A, Fennell T, Ruan J, Homer N, et al. The Sequence Alignment/Map format and SAMtools. Bioinformatics. 2009;25(16):2078-9.
25. Anders S, Pyl PT, Huber W. HTSeq--a Python framework to work with high-throughput sequencing data. Bioinformatics. 2015;31(2):166-9.
26. Wingett SW, Andrews S. FastQ Screen: A tool for multi-genome mapping and quality control. F1000Res. 2018;7:1338.
27. Gudbjartsson DF, Bjornsdottir US, Halapi E, Helgadottir A, Sulem P, Jonsdottir GM, et al. Sequence variants affecting eosinophil numbers associate with asthma and myocardial infarction. Nat Genet. 2009;41(3):342-7.
